# Targeting Early Atherosclerosis: A Focus on Oxidative Stress and Inflammation

**DOI:** 10.1155/2019/8563845

**Published:** 2019-07-01

**Authors:** Patricia Marchio, Sol Guerra-Ojeda, José M. Vila, Martín Aldasoro, Victor M. Victor, Maria D. Mauricio

**Affiliations:** ^1^Department of Physiology, Faculty of Medicine and Odontology, Universitat de Valencia and Institute of Health Research INCLIVA, Valencia, Spain; ^2^Service of Endocrinology, University Hospital Doctor Peset, Foundation for the Promotion of Health and Biomedical Research in the Valencian Region (FISABIO), Valencia, Spain

## Abstract

Atherosclerosis is a chronic vascular inflammatory disease associated to oxidative stress and endothelial dysfunction. Oxidation of low-density lipoprotein (LDL) cholesterol is one of the key factors for the development of atherosclerosis. Nonoxidized LDL have a low affinity for macrophages, so they are not themselves a risk factor. However, lowering LDL levels is a common clinical practice to reduce oxidation and the risk of major events in patients with cardiovascular diseases (CVD). Atherosclerosis starts with dysfunctional changes in the endothelium induced by disturbed shear stress which can lead to endothelial and platelet activation, adhesion of monocytes on the activated endothelium, and differentiation into proinflammatory macrophages, which increase the uptake of oxidized LDL (oxLDL) and turn into foam cells, exacerbating the inflammatory signalling. The atherosclerotic process is accelerated by a myriad of factors, such as the release of inflammatory chemokines and cytokines, the generation of reactive oxygen species (ROS), growth factors, and the proliferation of vascular smooth muscle cells. Inflammation and immunity are key factors for the development and complications of atherosclerosis, and therefore, the whole atherosclerotic process is a target for diagnosis and treatment. In this review, we focus on early stages of the disease and we address both biomarkers and therapeutic approaches currently available and under research.

## 1. Epidemiology

Cardiovascular diseases (CVD) are the leading cause of mortality in the Western population [1]. Atherosclerosis is considered a progressive inflammatory systemic disease affecting mainly the wall of large and medium arteries, such as the aorta, carotid, and coronary arteries [2, 3], at sites prone to low, turbulent, or oscillatory shear stress, like branches, curvatures, or bifurcations [4]. Although clinically relevant lesions become evident in middle-aged adults, it has been demonstrated that fat accumulation (known as fatty streaks) begins in early childhood [5]. The latency period is long, and clinical manifestations become evident several years later [6]. Cardiovascular (CV) risk factors such as hypercholesterolemia, hyperglycaemia, obesity, hypertension, smoking, and aging promote vascular inflammation and endothelial activation [7–9]. Controlling these factors reduces the risk of acute vascular complications and death from CVD [1, 7]. In accordance with the latest report of the World Health Organization (WHO), deaths from noncommunicable diseases account for almost 74% and they are mainly attributed to CVD [10]. The incidence of target organ damage associated to CVD increases with age, and gender studies show global higher incidence in men for stroke and coronary artery disease (CAD) [10]. The global mortality rate for CVD has significantly decreased in the last years; however, stroke and CAD remain the leading causes of mortality for CVD in adults [6, 10].

Oxidation of low-density lipoprotein (LDL) cholesterol is crucial in the development of atherosclerosis, and low LDL levels reduce the risk of major events in patients with CVD [6]. Despite that macrophages have low affinity for nonoxidized LDL, reducing LDL levels prevents oxidation, as recognized by European and American cardiac societies in their guidelines [11]. Besides the importance of this process, oxidation of LDL is not the sole initiator of inflammation, as the imbalance between oxidants and antioxidants is also important for the process of atherogenesis.

The control of the risk factors is the main cost-effective available measure for preventing major events associated to CVD [10]. There are promising therapies to attack the formation of the atheroma plaque. Therefore, with the aim to summarize the current knowledge on the initiation of the atherosclerotic process, in this paper, we review the early markers of atherosclerosis and we address the main therapeutic targets for preventing atheroma formation at its very initial stages focusing on inflammation, oxidative stress, endothelial dysfunction, and the interaction between platelets and endothelium.

## 2. The Vascular Wall: Structure and Function

**Figure 1:**
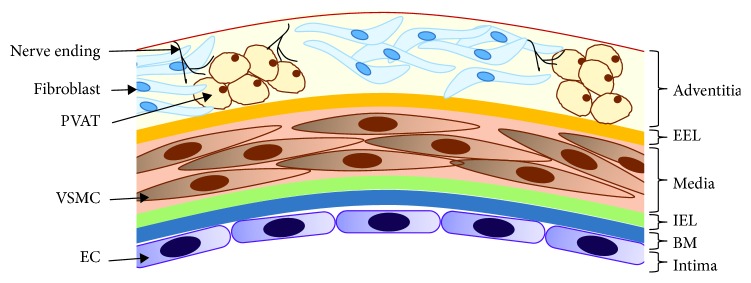
The structure of the vascular wall. PVAT: perivascular adipose tissue; VSMC: vascular smooth muscle cells; EC: endothelial cells; EEL: external elastic lamina; IEL: internal elastic lamina; BM: basement membrane.

The structure of the vascular wall is illustrated in Figure 1. The intima is the inner coat of the vessel, formed by one layer of endothelial cells (ECs) that lies on the basement membrane (BM) via adhesion molecules [12] and separated from the media by the internal elastic lamina (IEL) [13]. The endothelium is a semipermeable barrier with intercellular junctions (tight junctions) that regulates the passage of molecules through the vascular wall [14–16]. Among the several properties attributed to the endothelium, the most important are the maintenance of vascular tone by the release of vasodilator and vasoconstrictor factors, the preservation of an antithrombotic state, the participation in both immune and inflammatory responses and haemostasis, and the regulation of vascular permeability [14]. Therefore, the endothelium plays an important role in vascular homeostasis. In addition, most of the atheroprotective properties of the endothelium are attributed to nitric oxide (NO) [8, 17].

The ECs express phenotypic variation within the vascular tree. Actually, this variation that can evoke different biological responses to the same kind of stimulus can also affect adjacent or nearby cells [14]. The BM, part of the extracellular matrix (ECM), is mostly composed of different types of collagen, laminins, nidogens, proteoglycans, fibronectin, and von Willebrand factor (vWF) and provides mechanical support and an environment for cell interaction and molecule activity [12].

The media are primarily composed of vascular smooth muscle (VSM) cells and the ECM. VSM cells show two phenotypes: contractile and secretory. In general, the contractile phenotype is the most abundant and can be converted to a secretory phenotype under pathophysiological stimuli such as inflammation [12]. The most common is that, under a variety of stimuli, VSM cells mediate vascular contraction and relaxation by a calcium-dependent mechanism [18]. The secretory phenotype, less differentiated, is able to proliferate, migrate, produce, and secrete ECM proteins [12]. Most of the ECM of this tunica is produced by the VSM cells. ECM not only provides structural and mechanical support but also prompts to cellular interactions. It also acts as a physical barrier, and its integrity is crucial for the normal functioning of the vessel, as its disruption triggers multiple cellular responses [3, 12]. It is mainly composed by elastin fibers, which besides having a structural function are involved in the regulation of the proliferation of VSM cells. Other components of the ECM are collagen fibers, mainly types I and III, found between elastic fibers [12].

The external elastic lamina separates the media from the outer coat, the adventitia. It is a complex coat formed by fibroelastic connective tissue, where the most predominant component of ECM is the proteoglycan versican, which interacts with other components of the ECM participating in the compressible properties of the vascular wall and connecting the blood vessel with the surrounding connective tissue [12]. The adventitia is actively involved in both immune and inflammatory responses, vascular development and remodelling, cell signalling, and regulation of vascular tone. Besides ECM, it contains the vasa vasorum, perivascular adipose tissue (PVAT), nerve endings, lymph vessels, tertiary lymphoid structures, and different types of cells such as fibroblasts, macrophages, dendritic cells (DCs), T and B cells, and mast and plasma cells, giving this coat a key role in the regulation of vascular wall function [3, 13, 19, 20]. Adventitial cells respond to stimuli by producing cytokines, chemokines, reactive oxygen species (ROS), and remodelling substances. Fibroblasts are the most abundant cell type that predominantly produce fibrillar collagen (mainly types I and III). Fibroblasts not only have a mechanical function by producing ECM but are also involved in the first vascular responses to a variety of stimuli, acting as sensors of pathophysiological processes, such as inflammation and proliferation [21]. Macrophages and DCs also participate throughout the immune response stages. Activated fibroblasts differentiate into myofibroblasts, which are involved in the development of vasculopathies or physiological vascular remodelling by producing collagen and other ECM products. This process is regulated by ECM molecules including endothelin-1 (ET-1), angiotensin-II (Ang II) or interleukins (IL), and cytokines and adhesion molecules. In pathological conditions, myofibroblasts help to maintain a contractile vascular tone and migrate to the other coats, contributing to abnormal vascular remodelling. For instance, matrix metalloproteinases (MMPs), which are involved in the degradation of the ECM components (such as BM collagen, interstitial collagen, fibronectin, and various proteoglycans), in pathological conditions are upregulated and take part in the fibroblast/myofibroblast movement toward the other coats, explained by the “outside-in” process participating in the initial phases of inflammation and vascular remodelling [21–23].

The vasa vasorum is a network of microvessels that supplies nutrients and oxygen and drains wastes of large blood vessels. It regulates its own vascular tone which can be altered in inflammatory processes [24]. Vasa vasorum is involved in several vascular pathologies (like atherosclerotic plaque growth and complication) through expansion and neovascularization triggered by resident adventitial cells, such as macrophages and activated fibroblasts, by releasing inflammatory mediators and proangiogenic factors [21, 23, 24]. This involvement is consistent with the hypothesis that vascular inflammation progresses from the adventitia toward the media and intima, according to the “outside-in” process [25].

The perivascular adipose tissue is involved in the control of vascular tone of visceral arteries such as aorta, mainly through the release of adipokines such as adiponectin, which induces vasodilation by increasing NO bioavailability in human vessels [26]. The adipocyte-derived relaxing factor (ADRF) induces endothelium-independent vasodilation by opening voltage-dependent potassium channels (KCNQ channel family) in arterial smooth muscle cells. The ADRF remains to be identified, but hydrogen sulfide (H_2_S) is one of the most likely candidates [19, 27]. Alterations in the paracrine function of ADRF seem to be related to cardiovascular and metabolic disorders. Besides, the PVAT receives sympathetic innervation and secretes other signalling molecules with vasoactive properties such as vascular endothelial growth factor (VEGF), Ang II, or ROS [19].

## 3. The Atherosclerotic Process

### 3.1. Mechanotransduction and Atherosclerosis

Very early in the atherosclerotic process, dysfunctional changes in the endothelium, such as an increased permeability to lipoproteins, are most evident near branch points and bifurcations. Flow in these areas is called disturbed flow and is characterized by recirculation and eddy currents. However, these changes are not present in the regions of the vasculature associated with laminar blood flow. Therefore, depending on the type of blood flow acting on the endothelium, it may induce a proatherogenic or antiatherogenic phenotype, thus explaining the nonrandom localization of atherosclerotic lesions. Laminar blood flow and sustained high shear stress protect against atherosclerosis; conversely, disturbed blood flow and the low and reciprocating shear stress related to are associated with atherosclerosis.

Mechanical forces applied on the endothelium, such as shear stress, increase in a circumferential stretch, or high intraluminal pressure, can modulate gene expression, structure, and function, thereby inducing changes in biochemical pathways. This process is known as mechanotransduction [28] and has been implicated in the initiation of atherosclerotic lesions [29]. Mechanotransduction induces conformational changes in the cell cytoskeleton, in the cell-cell and the cell-ECM adhesion complexes. VEGF receptor, integrins, or glycocalyx can be disrupted by shear stress, serving as mechanosensors [30].

Laminar blood flow downregulates atherogenesis-related genes such as monocyte chemotactic protein-1 (MCP-1) and upregulates antioxidant and growth arrest genes in ECs. Conversely, disturbed flow at branch points of the arterial tree causes induction of MCP-1 and enhances monocyte infiltration in ECs [31]. Moreover, EC turnover is accelerated in areas with disturbed flow associated to low shear stress, probably due to the release of p21 suppression of cyclin-dependent kinase activity via G0/G1-S transition [32]. Accelerated cell turnover is likely to lead to an enhanced macromolecular permeability, increasing lipid uptake at regions of disturbed flow, which in turn would lead to an atherosclerotic phenotype [33].

Reciprocating flow can induce the expression of intercellular adhesion molecule-1 (ICAM-1), E-selectin, ET-1, and an increase of oxidative stress in ECs [29] by upregulation of gp91phox and NADPH oxidase 4 (Nox4) expression [34]. Vasodilator factors such as NO or prostacyclin are not upregulated by reciprocating flow [35], whereas the expression of VEGF is increased in response to low shear stress, leading to greater endothelial permeability [36]. Disturbed flow activates sterol regulatory element-binding protein- (SREBP-) mediated gene expression and hence leads to enhanced LDL uptake and lipid synthesis [37]. However, in physiological conditions, steady laminar flow has little effect on E-selectin and ICAM-1 expression or even causes a downregulation of ET-1 and vascular cell adhesion molecule-1 (VCAM-1) and increases NO and prostacyclin synthesis [29]. Therefore, steady laminar flow with high shear stress has a protective effect against atherosclerosis, whereas disturbed flow induces a proatherogenic phenotype, thereby explaining the focal nature of atherosclerosis along the vascular tree and highlighting the importance of the local hemodynamic environment.

Kruppel-like factors 2 and 4 (KLF2 and KLF4) are two important mechanosensitive transcription factors (MSTFs) upregulated after exposure to unidirectional laminar flow. KLF2 downregulates proinflammatory, prothrombotic, and vasoconstrictive genes, such as VCAM-1, MCP-1, E-selectin, ET-1, and plasminogen activator inhibitor-1 (PAI-1). Downstream target genes for KLF2 include endothelial nitric oxide synthase (eNOS) or thrombomodulin (THBD). The gene expression regulated by KLF4 overlaps with that regulated by KLF2. The protective roles of KLF2 and KLF4 have been demonstrated in experimental models of atherosclerosis (ApoE-deficient and LDL receptor-deficient mice), where deficiency of KLF2 and KLF4 accelerates the process [38].

Nuclear factor- (erythroid-derived 2-) like 2 (NFR2) is a MSTF which is activated in response to unidirectional laminar flow, playing an important role in EC adaptation to oxidative and nitrosative stress [39]. By contrast, nuclear factor-*κ*B (NF-*κ*B), activator protein 1 (AP-1), hypoxia-inducible factor 1a (HIF-1a) or Yes-associated protein (YAP), and transcriptional coactivator with PDZ-binding motif (TAZ) are suppressed by unidirectional laminal flow [40]. Otherwise, disturbed flow has opposite effects on these MSTFs.

YAP and TAZ, two effectors of the Hippo pathway, have been identified as MSTFs, and some studies show their role in the development of atherosclerosis. Wang et al. [41] found that endothelial YAP/TAZ inhibition suppresses c-Jun N-terminal kinase (JNK) signalling, decreases the inflammation process, and reduces monocyte infiltration, thus retarding atherogenesis. YAP knockdown was also shown to retard plaque formation in ApoE^−/−^ mice. The authors also reported that statins inhibit YAP/TAZ transactivation; however, simvastatin was not able to constitutively suppress YAP/TAZ in ECs. In addition, they indicate that unidirectional shear stress activates the integrin-G*α*_13_-RhoA-YAP pathway, which produces YAP phosphorylation and suppression, reducing plaque formation [41]. In the same line, Wang et al. observed that YAP/TAZ activation via biomechanical stretching regulates critical aspects of the human umbilical arterial smooth muscle cell (HUASMC) phenotypic switch, as YAP/TAZ knockdown attenuated the stretch-induced proliferative and proinflammatory phenotypes. Moreover, they reported that treatment with atorvastatin suppressed YAP/TAZ expression [42].

Considered together, these studies reveal that MSTFs represent promising therapeutic targets for the prevention of atherosclerosis. Due to it being beyond the scope of our paper, we recommend the recent review by Niu et al. which present a comprehensive overview of the role of MSTFs in atherosclerosis [40].

Therefore, the ECs of vascular areas exposed to disturbed shear stress (low, turbulent or oscillatory shear stress) exhibit an increased expression and activity of proinflammatory, proapoptotic, vasoconstrictor, and oxidant factors and a reduction in protective factors. These atherogenic properties of disturbed shear stress promote endothelial injury and trigger focal plaque formation [4]. In other words, disturbed shear stress promotes the endothelial atherogenic phenotype, whereas laminar and high shear stress induces the atheroprotective one.

### 3.2. Atheroma Plaque Formation

The atherosclerotic process involves the concurrence of systemic risk factors with disturbed shear stress and a vascular wall biological response [4].

The endothelial atherogenic phenotype has an increased permeability to circulating LDL, and their accumulation in the tunica intima is the first step in plaque formation [2]. LDL are exposed to oxidation, producing oxidized LDL (oxLDL), acting as damage-associated molecular patterns (DAMPs), damaging the endothelium, and triggering the inflammatory process by binding to pattern recognition receptors (PPRs) [2, 43, 44]. Humoral and cellular elements from the media and adventitia contribute to the progression of the disease, connecting with the intima through the fragmentation of the IEL under the atheroma and through the vasa vasorum that gives rise to the microvasculature of the plaque [3].

Activated ECs from damaged endothelium express cytokines, chemokines, and adhesion molecules such as MCP-1, ICAM-1, VCAM-1, E-selectin, and P-selectin, attracting circulating monocytes toward the atherosclerotic lesion, inducing the maturation of monocytes into proinflammatory macrophages (M1 phenotype) [43, 45, 46].

In normal conditions, macrophages regulate lipoprotein metabolism by controlling LDL and cholesterol content in order to maintain cholesterol homeostasis [2]. Macrophages express on their surface scavenger receptors (SR) such as CD36, SR-A1, and lectin-like oxLDL receptor-1 (LOX-1) that bind to oxLDL allowing the uptake of these proteins into the cell [46]. Macrophages express enzymes such as acyl coenzyme A: cholesterol acyltransferase-1 (ACAT1), responsible for the formation of cholesterol esters, and hydrolases, and lipases that cleave cholesterol esters into free fatty acids and cholesterol for storage [46]. Free cholesterol is also carried outside the cell by the ATP-binding cassette transporters ABCA1 and ABCG1 and the scavenger receptor SR-BI [46]. However, this regulation is altered in atherosclerosis, being that upregulated enzymes enable cholesterol accumulation and downregulated the expression of cholesterol transporters out of the cell [46]. In this sense, foam cells are the result of an unregulated accumulation of oxLDL and cholesterol esters within the macrophages located in the intima in response to activated ECs by inflammation. In a lesser degree, foam cells are derived from transformed smooth muscle cells [46].

The initial innate immune response is followed by an antigen-specific adaptive immune response involving different types of T and B cells [44]. Adaptive immunity has the capacity to selectively recognize molecules through surface B cell (BCR) and T cell (TCR) receptors. Moreover, T cells have CD4, CD8, or CD3 as coreceptors, which associated with TCR enable intracellular signalling transduction upon the recognition of an antigen-presenting cell [47]. In this regard, after having recognized the antigen, naïve T cells are primed into the different T cell types, whether into the plaque or into the lymphoid organs [48]. T helper 1 (Th1) is the most frequent T cell involved in the atherosclerotic process. Macrophage-derived IL-12 and IL-18 induce Th1 cell differentiation, responding to oxLDL stimuli by secreting further tumour necrosis factor *α* (TNF-*α*) and interferon *γ* (IFN-*γ*), a powerful inductor of atherosclerosis at the different stages of the process. [44, 47, 48]. Th2 plays a minor role, but it seems to be protective, secreting interleukins that inhibit Th1 cells and induce B1 cells or M2 macrophages. However, ApoE^−/−^/IL-4^−/−^ mice showed a significant reduction in plaque size, raising the suggestion that Th2 could also be atherosclerotic [49]. The role of Th17 cells and NKT are not yet fully understood, but they seem to possess both pro- and antiatherogenic properties. Regulatory T (Treg) cells act as atheroprotective cells by secreting IL-10 and transforming growth factor *β* (TGF-*β*), playing an immunomodulatory role [47]. B cells mainly function as antigen-presenting cells for T cells and antibody secretors, modulating immune response. B1 cells have atheroprotective effects by blocking oxLDL uptake by macrophages whereas B2 cells aggravate atherosclerosis by secreting autoantibodies and cytokines that trigger Th1 cells and macrophage activation [44]. In the atherogenic process, Th1 just as Th17, Th2, and B cells increase whereas Treg progressively decreases [50]. Most of the T cells in the atherosclerotic plaque are CD4^+^ Th1, thus predominating the proatherogenic type, followed by CD8^+^ and, to a lesser extent, Th2, Treg, Th17, cells and NKT cells. All subtypes of Treg are atheroprotective, and forkhead box P3 (Foxp3^+^) Treg, and type 1 regulatory T cells (Tr1) act by inducing IL-10 and TGF-*β* and cell-mediated inhibition.

At this point, if not degraded, foam cells accumulate and, together with macrophages inside the plaque, exacerbate the inflammatory signalling. This is achieved by releasing chemokines and cytokines that include IL-1, IL-6, TNF-*α*, IFN-*γ*, and, by producing ROS, growth factor and VSM cell proliferation, thus accelerating the development of atherosclerosis [46].

Specifically, the atheroma plaque is composed of a necrotic lipid core, which is a result of dead foam cells, circulating inflammatory and immune cells (such as T cells, macrophages, and mast cells), endothelial and smooth muscle cells, detritus and connective tissue elements, and a fibrous cap surrounding the plaque [2].

**Figure 2:**
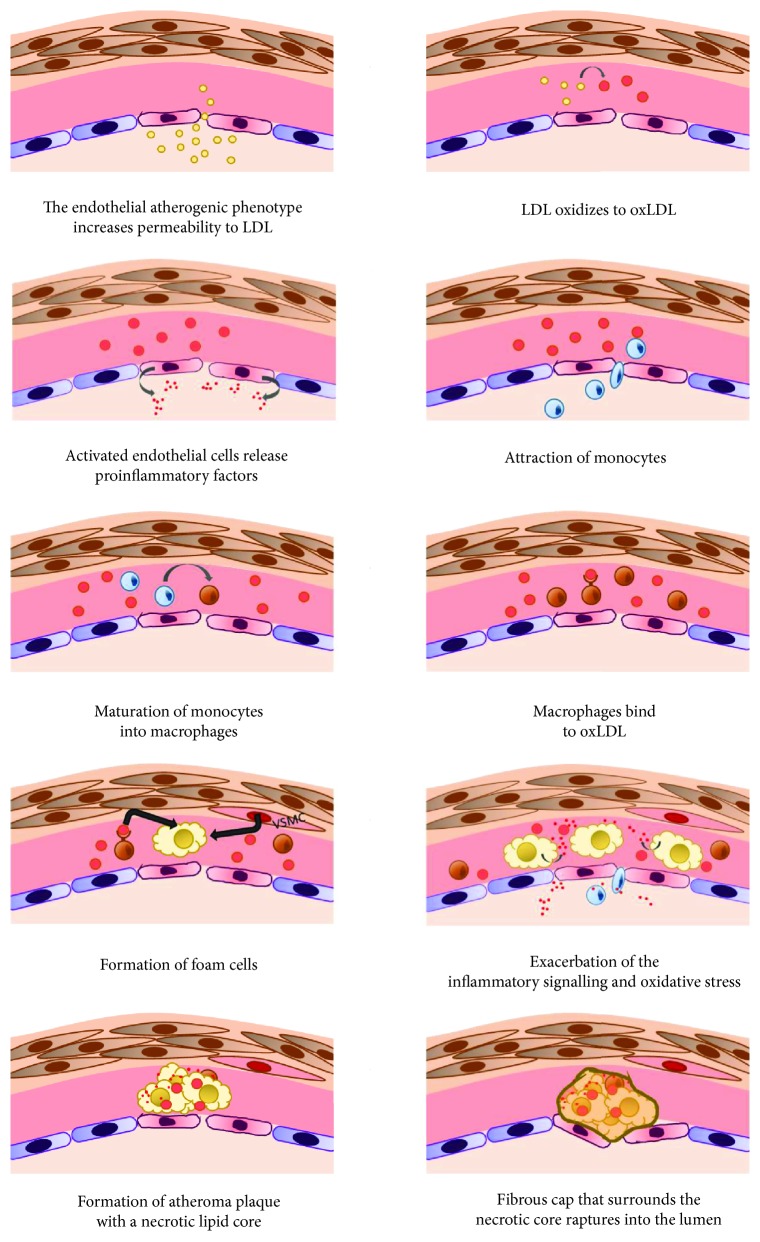
Atheroma plaque formation steps from endothelial dysfunction to rupture into the vascular lumen.

Inflammation and immunity are actively involved in the genesis and complications of atherosclerosis [3, 45], and inflammatory biomarkers are independent risk factors for cardiovascular events (CVE) [45]. Accordingly, thrombotic complications of atherosclerosis occur when the fibrous cap that surrounds the necrotic core ruptures into the lumen of the vessel [2]. The fibrous cap is destroyed by the action of proteolytic enzymes and the intense immune and inflammatory activity in the plaque, transforming the stable plaque to unstable and therefore increasing the risk of plaque rupture and thrombosis [2].  Figure 2 shows the atheroma plaque formation.

### 3.3. Oxidative and Nitrosative Stress in Early Atherosclerosis

Oxidative and nitrosative stress is characterized by an imbalance between the oxidant and antioxidant systems, resulting in an increase of reactive oxygen and nitrogen species (RONS). The vascular wall has oxidant systems such as xanthine oxidase [51], mitochondrial respiratory chain enzymes [52], lipoxygenases [53], uncoupled eNOS [54], NADPH oxidases (Nox) [55], and antioxidant systems, including superoxide dismutase (SOD), catalase, glutathione peroxidases, paraoxonases (PON), thioredoxin system, and peroxiredoxins [56].

Nox is considered the main source of RONS at the vascular wall. It reduces O_2_ to superoxide anion (O_2_^−^) [55]. ECs express Nox2 [57], Nox4, and Nox5 [58], whereas VSM cells express Nox1 [59], Nox4, and Nox5 [58]. The most abundant isoform at a vascular level is Nox4 [58, 60], playing a controversial role as a result of its both pro- and antiatherogenic functions. Nox4 releases more hydrogen peroxide (H_2_O_2_) than O_2_^−^ [61]; thus, the amount of peroxynitrite (ONOO^−^) formed is lower, and consequently, NO bioavailability is preserved [62–67]. Other studies show that an increase in Nox4 activity undermines vascular function in some diseases, such as diabetic cardiomyopathy [68].

Xanthine oxidase uses molecular O_2_ as an electron acceptor and forms O_2_^−^ and H_2_O_2_ generating uric acid, which triggers foam cell formation. A population-based study concluded that allopurinol, a xanthine oxidase inhibitor, had a role in reducing the risk of coronary artery disease [69]. Studies using experimental mouse models of atherosclerosis demonstrated an attenuation of the atherogenic process using xanthine oxidase inhibitors [70]. Expression of endothelial xanthine oxidase increases with elevated levels of Ang II and oscillatory shear stress, contributing to vascular dysfunction [51, 71].

Mitochondrial respiratory chain enzyme dysfunction leads to an increased ROS production. Experiments involving the deletion of antioxidant systems in ApoE^−/−^ mice suggest a role for mitochondrial ROS in atherogenesis [72].

Lipoxygenases use arachidonic acid to form hydroperoxides. The types related to atherogenesis are 5-lipoxygenase and 12/15-lipoxygenases, as they are able to activate Nox in VSM cells [73]. End products of lipoxygenases are leukotrienes, which have proinflammatory effects and release cytokines and MMPs [74].

The increased activity of Nox leads to eNOS uncoupling, reducing NO bioavailability and leading to endothelial dysfunction. Uncoupled eNOS exhibits Nox activity and produces O_2_^−^, thereby aggravating the vascular oxidative stress. The main causes of eNOS uncoupling are related to oxLDL, deficiency of L-arginine or tetrahydrobiopterin (BH4), eNOS S-glutathionylation [52], and hyperglycaemia [75, 76]. Therefore, nitric oxide synthases (NOS) play both an antioxidant and prooxidant role in atherosclerosis. eNOS is constitutively expressed in ECs and produces NO that inhibits LDL oxidation, leukocyte adhesion and migration, VSM cell proliferation, and platelet aggregation [77]. eNOS deletion in experimental models such as ApoE^−/−^ mice increases the atherosclerotic process [78]. Neuronal NO synthase (nNOS), expressed in central and peripheral nerve cells and in the vascular wall, contributes to vasodilation and is considered antiatherogenic. Conversely, inducible NOS (iNOS) induced by inflammation, oxidative stress, and sepsis is proatherogenic, likely due to the formation of peroxynitrite (ONOO^−^), thus increasing nitrosative stress [79]. Hence, iNOS activation can lead to deficiency of BH4 and thereby eNOS uncoupling [53].

Among antioxidant systems, three isoforms of SOD neutralize O_2_^−^ to form O_2_ and H_2_O_2_. SOD1 is located in the cytoplasm and the inner mitochondrial membrane, SOD2 is found in the mitochondrial matrix, and SOD3 is extracellular. Although SOD reduces O_2_^−^, it produces H_2_O_2_ and may enhance oxidative stress if there is no sufficient enzyme downstream [52]. Therefore, catalase that converts H_2_O_2_ to water and oxygen is necessary to diminish the damage induced by oxidative stress. Indeed, SOD1 overexpression alone may increase the extent of atherosclerosis; however, overexpression of catalase, in addition to SOD1, reduces atherosclerosis in ApoE^−/−^ mice [80].

Glutathione peroxidase (GPx) represents the major antioxidant system within many cells, reducing H_2_O_2_ and lipid hydroperoxides to water and their corresponding alcohols, where reduced glutathione (GSH) is the main electron donor. GPx oxidizes GSH to form glutathione disulfide (GSSG), a reaction that is reversed by the glutathione reductase, a NADPH-dependent enzyme [81]. The deficiency in glutathione peroxidase in mice produces an increase of oxLDL-induced foam cell formation [82], and human atherosclerotic lesions have been related to a decreased glutathione antioxidant function [83].

The paraoxonase family is composed of three members, where PON2 and PON3 are expressed in the vascular wall. They exert atheroprotective and anti-inflammatory properties by degrading H_2_O_2_, thus preventing lipid peroxidation [52]. Studies in mice have demonstrated their protective role against atherosclerosis by reducing oxidative stress [84]. A low expression of paraoxonases was found in the VSM cells of human atherosclerotic plaques, thus suggesting their protective role by preventing mitochondrial O_2_^−^ formation [85].

The thioredoxin (TRX) system, integrated by thioredoxin, NADPH, and thioredoxin reductase, regulates the equilibrium between protein dithiol and disulphide. The system provides electrons to peroxiredoxins in order to remove RONS, and the reduced TRX peroxidase scavenges H_2_O_2_ [86]. In ECs, TRX is a ROS-inducible protein, whereas in VSM cells, it is related to cell proliferation by a ROS-independent mechanism [87]. TRX increases in response to iNOS activation during plaque formation in rats, thus representing a mechanism against RONS and atherosclerosis [88]. Moreover, downregulation of thioredoxins is related to early stages of atherosclerosis by causing an endothelial prothrombotic phenotype in mouse models [89].

Peroxiredoxins are a family of proteins that use TRX as electron donor to regulate the levels of H_2_O_2_. Their function depends on the reduced forms of TRX and glutathione [90]. Peroxiredoxin 4 scavenges intracellular ROS from the endoplasmic reticulum, and it has been demonstrated that oxidative stress and endoplasmic reticulum stress contribute to the onset of inflammation in vascular diseases such as atherosclerosis [91].

### 3.4. Crosstalk between Oxidative Stress and Inflammation in Early Atherosclerosis

NF-*κ*B forms a family of inducible transcription factors regulating genes that participate in immune and inflammatory responses as well as in the cell cycle. It is composed of NF-*κ*B1 or p50, NF-*κ*B2 or p52, RelA or p65, RelB, and c-Rel. They are located in the cytoplasm with their inhibitor, the I*κ*B family, which includes I*κ*B*α* and the I*κ*B*α*-like proteins p105 and p100, precursors of NF-*κ*B1 and 2, respectively. Two pathways, canonical and noncanonical, intervene in the NF-*κ*B activation. The most common is the canonical. It is activated by a variety of stimuli such as cytokines, microbes, or stress, which interact with receptors on the cell surface such as cytokine receptors, PPRs, and TNF receptor (TNFR) superfamily members, as well as TCR and BCR. Then, the transforming growth factor-*β*-activated kinase 1 (TAK1) activates the I*κ*B kinase IKK that phosphorylates the I*κ*B*α*, inducing the degradation of the I*κ*B*α* and the translocation of the activated NF-*κ*B group, mainly the p50/RelA and p50/c-Rel, into the nucleus. The noncanonical pathway acts as a support of the first one. It responds selectively to specific stimuli on the TNFR family (such as LT*β*R, BAFFR, CD40, and RANK). This interaction induces p100 phosphorylation by a NF-*κ*B-inducing kinase (NIK) together with IKK*α* and the consequent maturation of NF-*κ*B2 and the translocation of the NF-*κ*B2/RelB group into the nucleus. Uncontrolled activation of NF-*κ*B is involved in chronic inflammatory diseases. NF-*κ*B mediates proinflammatory gene induction and controls the activation, differentiation, and function of inflammatory T cells and regulates inflammasomes [92].

Innate immune cells, including macrophages, dendritic cells, and neutrophils, express PRRs (Toll-like receptors (TLRs) or NOD-like receptors (NLRs)) that recognize molecules released by microbes or damaged/necrotic cells and tissues. PRRs trigger the activation of the canonical NF-*κ*B pathway and therefore the induction of proinflammatory mediators in the innate immune cells, which provoke the inflammatory response and also promote inflammatory T cell differentiation. The NF-*κ*B, induced by TLR signalling, is involved in the differentiation of macrophages on the M1 type, leading to the production of a myriad of inflammatory mediators involved in several inflammatory conditions. M1 macrophages are also involved in the differentiation of inflammatory T cells, including T helpers (Th) Th1 and Th17. Naïve T cells, mainly CD4^+^ Th, participate in adaptive immune response. A specific stimulus interacts with the TCR, inducing the canonical NF-*κ*B groups RelA and c-Rel and therefore aberrant T cell activation with the consequent inflammatory and autoimmune responses. NF-*κ*B also mediates CD4^+^ T cell differentiation. Th1 and Th17 participate in inflammatory responses, releasing mediators, such as IFN-*γ* and IL-17, respectively [92, 93].

The transcription of NF-*κ*B-dependent genes influences the levels of ROS in the cell, and in turn, the levels of NF-*κ*B activity are also regulated by the levels of ROS. ROS interact with NF-*κ*B both by inhibiting or stimulating at different sites on NF-*κ*B pathways, which in turn regulates cellular ROS levels. These interactions seem to be multiple and cell specific [92]. For instance, ROS are modulated by NF-*κ*B target genes as a means to stop cell damage induced by c-Jun N-terminal kinase (JNK). In this sense, crosstalk between NF-*κ*B and JNK downregulates JNK activation and therefore protects cells against ROS accumulation and toxicity [92, 94].

Furthermore, activation of NF-*κ*B pathways, by inducing the expression of both antioxidant and prooxidant proteins, influences ROS levels. The upregulation of antioxidant enzymes by ROS through NF-*κ*B protects cells from damage and death. On the other hand, NF-*κ*B activation contributes to ROS generation, as what happens in inflammation. Enzymes such as Nox, xanthine oxidase, iNOS, or nNOS are regulated by the NF-*κ*B pathway with the consequent production of RONS and peroxynitrites. Furthermore, COX-2 and other enzymes such as lipoxygenases form ROS as byproducts through the NF-*κ*B pathway, contributing to oxidative stress [92].

**Figure 3:**
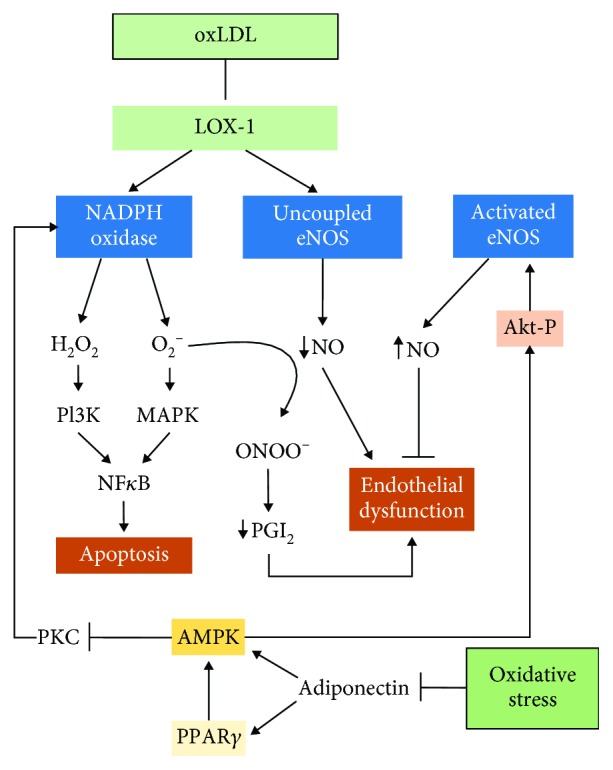
Effects of oxLDL and oxidative stress on endothelium. LOX-1 activation by oxLDL induces endothelial oxidative stress by increasing NADPH oxidase (Nox) activity and uncoupling eNOS. Oxidative stress activates NF-*κ*B through p38 mitogen-activated protein kinase (p38MAPK) and phosphatidylinositol 3-kinase (PI3K) transduction pathways initiating intranuclear apoptotic signal transduction. The formation of peroxynitrite (ONOO^−^) reduces nitric oxide (NO) and prostacyclin (PGI2) bioavailability leading to endothelial dysfunction. In addition, oxidative stress reduces PPAR*γ* activity and adiponectin levels. Both of them stimulate AMP-activated protein kinase (AMPK) which in turn upregulates eNOS activity through Akt phosphorylation (Akt-P). Moreover, AMPK is a negative regulator of Nox.

As already known, early atherosclerosis is characterized by oxidative stress and inflammation, which have a cyclical relationship, since the inflammatory process that tries to repair oxidative damage can induce more oxidative stress, thus resulting in endothelial dysfunction. We can assume that the starting point of atherosclerosis is a change from the EC phenotype toward the atherogenic phenotype, which leads to EC activation, increasing the permeability to LDL and its later oxidation, attracting circulating monocytes, primarily the Ly6Chi subtype, that turn into proinflammatory macrophages, which produce ROS mainly via Nox. ROS exert their actions mainly via NF*κ*B, which induces the synthesis of proinflammatory cytokines, such as TNF-*α*, which in turn activate NF-*κ*B [95]. Hence, due to the synergy between ROS and cytokines, ECs promote the synthesis of inflammatory factors and upregulate the expression of adhesion molecules, thus allowing neutrophils to transmigrate into the intima of an artery [96]. Neutrophils promote the accumulation of monocytes via neutrophil-derived cathelicidin [97]. Monocytes transform into proinflammatory macrophages that bind to oxLDL through their scavenger receptor causing them to release inflammatory cytokines and specific enzymes involved in the atherogenic process, such as carboxyl ester lipase or lipoprotein-associated phospholipase A2 [11]. The modified lipoprotein particles increase the expression of cell adhesion molecules (like VCAM-1, P-selectin, and E-selectin) on the ECs, leading to leukocyte recruitment (mainly monocytes and T cells) into the subendothelial space. With the interplay of chemoattractant proteins like MCP-1, eotaxin, and INF-*γ*, T cells and mast cells migrate into the intima and release cytokines, growth factors, and ROS that stimulate VSM cell migration and collagen deposition, thus initiating the development of the plaque [53]. In addition, oxLDL activates the cascade of local inflammation via NF*κ*B [98] through p38 mitogen-activated protein kinase (p38MAPK) and phosphatidylinositol 3-kinase (PI3K) transduction pathways [99, 100]. Chen et al. [101] proposed that the binding of oxLDL to LOX-1 activates Nox on the cell membrane, thereby increasing intracellular ROS, which acts as a second messenger and causes the activation of NF-*κ*B, which in turn initiates intranuclear apoptotic signal transduction pathways in ECs (Figure 3).

In addition, uric acid generated by xanthine oxidase triggers foam cell formation by increasing LOX-1 expression on macrophages and VSM cells and activates NLRP3 inflammasome and downstream inflammation [102]. The NLRP3 inflammasome is a cytoplasmic complex present in immune cells such as monocytes and neutrophils that detect dangerous signals [103]. Xanthine oxidase-mediated ROS formation has a proinflammatory effect by releasing inflammatory cytokines in macrophages from ApoE^−/−^ mice [70]. Again, both processes, oxidative stress and inflammation, come together.

Moreover, other pathways related to inflammation and which implicate peroxisome proliferator-activated receptor-*γ* (PPAR*γ*) and adiponectin are downregulated due to oxidative stress and we discuss them in Endothelial Dysfunction. Therefore, oxidative stress with excess ROS generation and oxidation of LDL plays an important role in inflammatory responses; both mechanisms exert a synergic effect on each other and alter vascular function and are critical in the development of atherosclerosis.

## 4. Targeting Early Atherosclerosis

As stated earlier, the event that initiates plaque formation is the accumulation of modified LDL in the intima and atherosclerosis is the result of the immune and inflammatory responses to this phenomenon. A study analyzing the human arterial tissue proteomics identified several vascular and plasma biomarkers related to early atherosclerosis [6] including TNF-*α*, insulin receptor, PPAR*α*, and PPAR*γ* protein networks, predictors of both development and site of atherosclerosis and CVD. In this regard, the early detection of the atherosclerotic process and, therefore, the prompt intervention to halt or reverse the immune and inflammatory processes would prevent thrombotic events from happening. Accordingly, the entire atherosclerotic process could become a rationale target for diagnostic and therapeutic research. In this sense, the main early targets are the endothelium, platelets, immune and inflammatory local and circulating cells, and mediators [104, 105]. In this section, we focus on endothelial dysfunction, the interaction between endothelium and platelets, and early biomarkers of atherosclerosis.

### 4.1. Endothelial Dysfunction

The main cause of endothelial dysfunction is the impaired bioavailability of NO [13]. NO is synthesized by the eNOS from L-arginine in the presence of molecular O_2_ and the following cofactors: BH4, reduced nicotinamide adenine dinucleotide phosphate (NADPH), heme, flavin adenine dinucleotide (FAD), flavin mononucleotide (FNM), and zinc [8]. NO diffuses to the luminal side of the wall preventing platelet adhesion and aggregation and also to VSM cells where it binds and activates the soluble guanylyl cyclase that catalyzes the conversion of guanosine-5′-triphosphate (GTP) to cyclic 3′,5′-guanosine monophosphate (cGMP) resulting in vasodilatation and inhibition of vascular remodelling [106]. Therefore, NO is a potent endogenous vasodilator that also prevents the expression of proinflammatory molecules such as NF-*κ*B and adhesion molecules ICAM-1 and VCAM-1 [16].

As stated earlier, Nox is the main source of RONS at the vascular wall. It reduces O_2_ to O_2_^−^ [55], which in turn interacts with NO to generate the very potent oxidant ONOO^−^, reducing the NO bioavailability and leading to endothelial dysfunction. Therefore, one of the main consequences of oxidative stress at the vascular level is the endothelial dysfunction, present at early atherosclerosis. Hence, to know the pathways implicated in this pathological process helps to develop drugs against incipient atherosclerosis.

ONOO^−^ is highly reactive and can easily cross biological membranes and interact with target molecules such as DNA, proteins, and lipids. ONOO^−^ oxidizes heme proteins, such as hemoglobin or cytochrome *c*, and iron sulfur-containing enzymes, such as eNOS, inducing their inactivation [79]. It also reacts with cysteine, oxidizing the thiol group, generating reactive products such as thiol radicals (RS^•^) that react with oxygen amplifying oxidative stress and with NO to produce nitrosothiols. The result of thiol oxidation is the inactivation of critical enzymes involved in cell metabolism and signalling [79]. ONOO^−^ can also induce vascular injury by means of proMMP activation by a glutathione- (GSH-) dependent mechanism [107]. One relevant mechanism of action of ONOO^−^ is through tyrosine nitration, altering protein function, enzyme activity, cell structure, and signalling. Tyrosine nitration is associated with an increased formation of ONOO^−^ and other RNS, as could be found in the progress of different diseases [79, 108]. Nitration and, consequently, inactivation of prostacyclin synthase (PGI_2_ synthase) in the arterial wall during inflammation by a CD40-dependent mechanism are involved in the development of endothelial dysfunction associated to atherosclerosis and other vascular diseases [79]. ONOO^−^ triggers lipid peroxidation in membranes, increasing membrane permeability. Of importance in the early phases of atherogenesis, LDL peroxidation facilitates LDL binding to scavenger receptors leading to the formation of foam cell [16, 79]. Moreover, excessive accumulation of circulating LDL creates a proinflammatory state that leads to a reduction in NO bioavailability [16, 109].

The role of oxLDL in the early stage of this process is of importance since the activation of its receptor (LOX-1) increases vascular oxidative stress and apoptosis leading to endothelial dysfunction [101]. LOX-1 is located in macrophages, VSM, and ECs. All of these cells are involved in the atherosclerotic process. LOX-1 activation promotes endothelial oxidative stress mainly through Nox activation and eNOS uncoupling [110] (Figure 3).

Other mechanisms related to oxidative stress can induce endothelial damage indirectly, for instance, by reducing PPAR*γ* activity or adiponectin levels. In this line, PPAR*γ* agonists can ameliorate oxLDL-induced endothelial dysfunction. Plenty of studies show a protective role against endothelial dysfunction through the activation of PPAR*γ* [111–113]. The experiments performed by Xu et al. [114] in rat microvascular EC culture demonstrated that PPAR*γ* agonists reversed oxLDL-induced endothelial dysfunction by stimulating AMP-activated protein kinase (AMPK), which is a serine/threonine protein kinase that upregulates the Akt/eNOS/NO pathway enhancing eNOS activity [115]. Consequently, the PPAR*γ*/AMPK/eNOS pathway could be a target for the treatment of atherosclerosis (Figure 3).

In addition, AMPK inhibits protein kinase C (PKC), which phosphorylates p47^phox^ and activates Nox in several types of cells, including vascular cells [116, 117] (Figure 3). Thus, AMPK has an important role in the prevention of vascular oxidative injury and hence endothelial dysfunction, since it is a negative regulator of Nox [118, 119]. Some AMPK activators, such as statins [120, 121], improve endothelial function and have antiatherogenic properties.

Moreover, oxidative stress negatively affects the levels of adiponectin [122]. The increasing importance of adiponectin is related to the fact that its levels decrease in some cardiovascular diseases such as obesity, type 2 diabetes, metabolic syndrome, or atherosclerosis [123]. This adipokine released by adipose tissue has insulin-sensitizing, anti-inflammatory, and antioxidant properties [124, 125]. There are two receptors for adiponectin, AdipoR1 and AdipoR2, both with antiatherogenic activity [126] through modulation of AMPK and PPAR ligand activity [127]. In ECs, adiponectin can downregulate the expression of adhesion molecules such as ICAM-1, which promotes monocyte adhesion to the vascular endothelium, by inhibiting TNF-*α*-mediated activation of NF-*κ*B [123, 128, 129]. Adiponectin can also increase the phosphorylation of eNOS at Ser1177 via AMPK, enhancing the eNOS activity [130]. Moreover, adiponectin inhibits the production of ROS induced by oxLDL in cultured ECs [131]. All these effects indicate that high levels of adiponectin could protect against atherosclerosis.

PPAR*α* or PPAR*γ* agonists increase the levels of adiponectin, such as some treatments for cardiovascular diseases like angiotensin-converting enzyme (ACE) inhibitors, angiotensin II receptor antagonists, or rosiglitazone in type 2 diabetes and statins in hypercholesterolemic patients [132, 133]. Moreover, some nutritional supplements, such as resveratrol and S-adenosylmethionine, exert their anti-inflammatory effects by increasing adiponectin levels [134, 135].

### 4.2. Platelet-Endothelium Interaction

In physiological conditions, the endothelium is protected from platelet adhesion and aggregation by releasing NO and prostacyclin and by degrading the platelet's ADP [16, 136]. If the endothelium is intact and healthy, circulating platelets remain in an inactivated state [137]. However, in inflammatory states, like that in the presence of cardiovascular risk factors, platelets adhere to the endothelium even in the absence of endothelial injury or platelet activation, as it has been demonstrated in humans and apolipoprotein-deficient mouse models [105, 136]. Nonetheless, when the endothelium is damaged, molecules from the ECM, such as collagen and vWF, and products derivate from platelets, such as thromboxane A_2_ (TXA_2_), ADP, and thrombin, trigger platelet activation [137]. Activated platelets aggregate and adhere to each other and to the subendothelium mainly through membrane glycoprotein receptors Ib and IIb/IIIa and interact with the endothelium contributing to endothelial activation, which is crucial for the initiation of the atherosclerotic process and pathologic thrombosis [2, 105, 137, 138].

**Figure 4:**
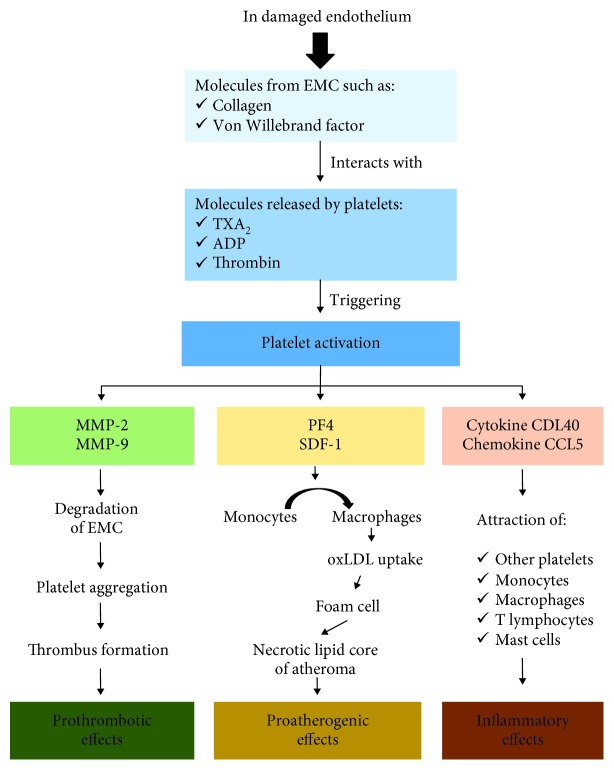
Prothrombotic, proatherogenic, and inflammatory effects of platelet activation. The platelet-endothelium interaction triggers platelet activation, considered as a critical point in all phases of atherosclerosis. ECM: extracellular matrix; MMP-2: matrix metalloproteinases 2; MMP-9: matrix metalloproteinases 9; PF4: platelet factor 4; SDF-1: stromal cell-derived factor-1.

Endothelial P- and E-selectins, adhesion molecule ICAM-1, vWF, and vitronectin allow the adhesion of platelets to the endothelium. Platelets also express receptors for cellular interaction such as P2Y12, P-selectin, and integrin, playing a role in thrombosis and inflammation [139]. They secrete and induce the release of cytokines and chemokines by other components in the vascular wall such as ECs. Platelet molecules, mainly through the chemokine CCL5 (also known as RANTES) and the cytokine CD40L, promote the attraction of other platelets and immune and inflammatory cells such as monocytes and macrophages, T cells, and mast cells to the plaque, amplifying the signalling cascade to further contribute to the progression of the disease [138]. PF4 (platelet factor 4 or CXCL4) and stromal cell-derived factor-1 (SDF-1) attract monocytes, favouring their maturation into macrophages, and stimulate oxLDL uptake, contributing to the formation of foam cells and the necrotic lipid core of the atheroma [138]. For this reason, platelet activation is considered critical in all phases of atherosclerosis, since platelets are involved in the development, progression, and complications of atherosclerosis [138]. On the other hand, platelets play a key role in plaque rupture as a result of the action of MMP-2 and MMP-9, degrading and exposing ECM to action of local factors that favour platelet aggregation and thrombus formation [138] by a CD40L-dependent process [138]. On the abovementioned basis, platelets have proatherogenic, proinflammatory, and prothrombotic effects (Figure 4). Platelet activation and platelet inflammatory biomarkers are elevated in most of the risk factors for CVD such as obesity, diabetes mellitus, or hypertension [139].

### 4.3. Biomarkers

The identification of biological markers of atherosclerosis is crucial for preventing the development, progression, and complications of the disease. Algorithms stratifying the cardiovascular risk are useful tools for detecting people who would benefit from primary and secondary prevention. However, some patients at risk fall in the lower categories [140]. For this reason, recent studies are focusing on additional screening methods, such as serum, genetic, and imaging markers of atherosclerosis, as extensively reviewed Tibaut et al. [141, 142].

The most widely recognized nonspecific biological marker of inflammation is high-sensitivity C-reactive protein (hsCRP). CRP is a plasma protein synthesized primarily by the liver and, to a lesser extent, by endothelial and atheroma cells [109, 141, 143]. It is an acute-phase reactant, released in response to acute inflammatory stimuli, and is considered a risk biomarker for cardiovascular events [144]. Yousuf et al. [143] reviewed CRP involvement in the atherosclerotic process. CRP is considered proatherogenic, acting at early and crucial stages of plaque formation. It binds oxLDL and triggers monocyte-macrophage activation and inhibits eNOS, impairing vasodilation and promoting endothelial dysfunction. Furthermore, in atherosclerosis, IL-6 produced by foam cells induces the production of small quantities of CRP. For clinical purposes, most trials found the cutting value of hsCRP ≥ 2 mg/l a reliable marker of inflammation and, therefore, a predictor of CV events [145, 146], although the CRP value for assessing the risk for CVD is limited [147].

Arterial wall calcification is a marker of atherosclerosis. A useful tool to assess it is the coronary artery calcium score (CAC) that measures the amount of calcium in the coronary artery wall by means of computed tomography (CT). CAC is a good predictor of CVE and is useful for the stratification of asymptomatic individuals and to detect those who will benefit from early treatment, such as subjects with moderate risk for CVD [142]. The Agatston score is used to measure wall calcium, which is standardized for coronary arteries. However, it is also used for another vascular trees but with great variability [148]. A CAC = 0 is considered very low risk for CVD whereas that >300-400 defines patients at high risk. Within the context of the Multi-Ethnic Study of Atherosclerosis (MESA), participants were followed during 10 years to evaluate the accuracy of biomarkers to predict CVD. Among the negative risk markers for CVD, a CAC = 0 was the most accurate to reclassify patients into a very low risk group and, therefore, less likely to benefit from preventive pharmacological treatment [147]. Coronary calcification has better correlation with CVE than other imaging methods, and having calcifications in other vascular beds increases the risk for CVE [149]. In this sense, another MESA study demonstrated that multisite atherosclerosis increased the risk for CVD, especially in subjects with risk factors. The authors also found that CAC is the strongest predictor marker for CVD [150]. Considering the concerns about the risk associated with radiation and the advantages of having an accurate stratification of CVD risk, it is important to establish which subjects will benefit for further explorations. In this regard, latest guidelines recommend CAC as a useful tool to refine risk assessment upward or downward in individuals with predicted risk of 5% to 20% for CVD [151].

Increased serum levels of IL-6 and IL-18, both proinflammatory cytokines involved in the atherosclerotic process, are also predictors of CV events [152–154], as we report later in Targeting Immunity and Inflammation of this review.

Other early inflammatory biomarkers for atherosclerosis include TNF-*α*, found useful in predicting CV events in the short term, as well as molecules involved in the initial phases of cell interaction and atheroma formation. In this regard, more studies are needed to elucidate the role of adhesion molecules such as VCAM-1, ICAM-1, E-selectin, and P-selectin as early markers of plaque formation [141].

Other potential useful markers could be T cells. Treg cells were decreased in patients with acute coronary syndrome [155, 156], but not in stable coronary disease compared to control patients [156]. However, a recent study in patients with stable coronary disease evidenced progression of atherosclerosis when CD4^+^IL10^+^ Treg cell blood levels were below 3.3% [157]. Furthermore, the Treg cell count was reduced in patients with mild carotid atherosclerosis [158]. Nevertheless, for the moment, the usefulness of T cells as biomarkers of early atherosclerosis remains to be elucidated.

Biomarkers of oxidative stress, such as MMPs, myeloperoxidase (MPO), oxLDL, or Nox could emerge as useful molecules to identify subclinical atherosclerosis once accurate screening methods become available [141].

microRNAs (miRNAs) are short noncoding RNA molecules involved in the regulation of gene expression. They participate in cell signalling and intracellular communication and seem to be involved in every step of the atherosclerotic process, as recently described Laffont et al. [159]. miRNAs control LDL and high-density lipoprotein (HDL) genesis and function, thus regulating the metabolism of lipoproteins. Of importance is the role of miRNAs and changes in their expression in the initiation of atherosclerosis, as they regulate endothelial and VSM cell function and macrophage activation. In this sense, miRNAs are promising biological markers and targets to early detection and to attack the atherosclerotic process from the initial stages [142, 159].

## 5. Preventing Atherosclerosis through Lifestyle Modification

As stated in the last ACC/AHA Guideline, to follow a healthy lifestyle (Life's Simple 7) is the most important measure to prevent atherosclerosis [151]. Most of the CVE in subjects without cardiovascular disease could be prevented by the avoidance of four unhealthy behaviors such as smoking, sedentary lifestyle, overweight, and nonsalutary diet and by the control of the following major risk factors for CVD: hypercholesterolemia, hypertension, and diabetes [151]. We briefly expose the main mechanisms by which these factors contribute to the development of atherosclerosis and the recommendations to control them. At least 80% of CVD could be prevented by elimination of health risks [1]. Psychological, social, and work stressors are also risk factors for CVD [1]. Therefore, multimodal approach is recommended. Detailed measures to prevent CVD are beyond of the scope of this review (see Piepoli et al. [1]).

### 5.1. Smoking

Smoking accounts for 10% of CVD cases [160]. Long-term smoking before the age of 50 years doubles the probability to die because of tobacco. Passive smoking is also a risk factor for CVD. Half of the deaths attributed to smoking are for CVD. Stopping smoking, whether actively or passively, is life saving and the most cost-effective measure to lower the risk for CVD [1].

Smoking damages endothelial function, increases oxidative stress, platelet activation, and inflammation, and promotes VSM cell proliferation and migration, contributing to atherosclerosis [1, 160].

Extensive vascular effects induced by smoking were already reviewed by other authors [160, 161]. Succinctly, smoking produces endothelial dysfunction through a variety of toxic chemical compounds, acting as a source and, at the same time, inducing oxidative stress. Tobacco decreases NO bioavailability, by increasing asymmetric dimethylarginine (ADMA) levels and uncoupling eNOS, by a mechanism that alters NADPH and xanthine oxidase enzymes [160, 161]. Vascular inflammation induced by smoking is related to a reduction in the expression of SIRT-4, decreasing I*κ*B expression, which results in an increased NF-*κ*B expression and, therefore, induction of inflammatory mediators. Furthermore, it increases vascular expression of a myriad of adhesive molecules, inflammatory chemokines, and cytokines promoting vascular reactions amplifying the inflammatory process that leads to atherosclerosis [160, 161].

In addition, tobacco alters serum lipids leading to a proatherogenic profile, increasing total serum cholesterol, very low-density lipoprotein (VLDL), LDL, and TG, and decreasing HDL. Furthermore, it promotes lipid peroxidation by peroxynitrite formation and by decreasing endogenous antioxidant defenses [160]. Nicotine, the main cigarette compound, is clearly involved in smoking-induced ROS. Furthermore, it has been demonstrated that nicotine promotes the switching from the contractile to the secretory VSM cell phenotype, via the ROS/NF-*κ*B signalling pathway [162].

### 5.2. Sedentary Lifestyle

A sedentary behavior is the awaking energy expenditure of ≤1.5 metabolic equivalents while in a sitting or reclining posture [151]. Sedentary lifestyle has become a major public health problem. It is an independent risk factor for atherosclerosis and CVD [151] and accounts for at least one third of deaths for coronary heart disease or type II diabetes [163, 164]. Half of deaths for CVD could be prevented by changing lifestyle. However, most people, including children and the young, spend more than 50% of their lives doing sedentary activities [163, 165, 166]. Several studies and meta-analysis evaluated the effects of sedentary behavior on CV risk [163]. Mortality rates were higher in sitting time > 8 h/day and low physical activity compared to those with sitting time < 4 h and high physical activity [167]. A worldwide study estimated that physical inactivity is responsible for a mean of 5.8% of the coronary artery disease, 7.2% of type 2 diabetes, and 9.4% of premature mortality [168]. A review and meta-analysis of 34 studies including a large population found a strong association with the total sedentary behavior and time watching TV and CVD mortality and type 2 diabetes. More than 6 h/day of total sitting and 3–4 h/day of TV viewing increased the risk of death for CVD [169].

In mice, physical inactivity induced vascular lipid peroxidation and ROS by increasing Nox expression and activity, leading to endothelial dysfunction and accelerated atherosclerosis [170]. Gratas-Delamarche et al. [165] reviewed the mechanisms involved in physiological derangements induced by physical inactivity, which are mainly related to insulin resistance, inflammation, and oxidative stress.

The reduced sensitivity to insulin in peripheral organs and tissues such as the liver, skeletal muscle, and adipose tissue alters glucose homeostasis by a reduction in glucose uptake, leading to hyperglycaemia and, therefore, insulin resistance and type 2 diabetes. Insulin resistance also alters lipid and protein metabolism. The increase in ROS and proinflammatory cytokines impairs the insulin signalling pathway and activates the NF-*κ*B pathway, perpetuating an inflammatory and oxidative environment, prolonging insulin resistance and, in a certain way, atherosclerosis. Even short terms of inactivity rapidly reduce insulin sensitivity in humans and experimental animals. In the endothelium, short-term bed rest alters microcirculation and decreases insulin sensitivity in humans, probably secondary to reduced local blood flow and therefore shear stress, leading to vascular dysfunction. Moreover, it reduces NO and increases ROS production and vasoconstrictor factors. In the skeletal muscle, inactivity contributes to early insulin resistance by altering insulin signalling pathways. It decreases glucose tolerance and uptake, Akt phosphorylation, and IRS and glucose transport type 4 (GLUT4) levels. Furthermore, muscle also develops a proinflammatory and oxidative environment and lipid peroxidation.

Sedentary behavior contributes to ectopic fat accumulation, such as visceral adiposity, considered a trigger for adipose tissue dysfunction, which involves increased ROS production by a Nox4 mechanism and the onset of inflammatory response by attracting and activating the M1-macrophage phenotype, by producing inflammatory mediators such as TNF-*α* and IL-6 and adipokines, besides reducing insulin sensitivity and secretion. Moreover, adipose tissue dysfunction also alters lipid metabolism and lipid peroxidation [165].

As reviewed by other authors [171, 172], in humans, sedentary behavior leads to metabolic dysfunction, with elevated plasma triglyceride levels and decreased HDL levels and insulin sensitivity, partly secondary to reduced lipoprotein lipase activity. Furthermore, sedentary behavior alters glucose transport by decreasing muscle glucose transport (GLUT) protein content, which is involved in glucose uptake and therefore glucose tolerance. In vascular vessels, sedentary behavior reduces vascular blood flow and increases blood pressure and the arterial diameter. Moreover, it impairs endothelium-dependent vasodilation and causes EC damage. Finally, effects of sedentary time are independent of levels of physical activity in the majority of studies. Sedentary behavior predisposes to overweight, obesity, metabolic syndrome, hypertension, type 2 diabetes, acute coronary syndrome, and abnormal tolerance to glucose [172].

Considering all the above, one of the most effective nonpharmacological approaches to prevent atherosclerosis and CVD is physical activity. Even small amounts of activity have protective results, and performing regular physical activity reduces the risk of CVD and all-cause and CVD mortality by 20-30% [1, 163, 164], even in the presence of risk factors for CVD [173]. At blood vessels, physical activity reduces vascular resistance and augments shear stress, eNOS expression, and NO bioavailability, which, altogether, enhances vasorelaxation and organ blood flow and reduces plaque formation and instability. At the metabolic level, physical activity increases HDL, insulin sensitivity, and glucose uptake and reduces VLDL, LDL, TC, and TG [173].

However, at least 40% of the benefits induced by exercise cannot be explained only by the control of the risk factors. They are also related to the direct and repetitive effects of exercise on the vascular wall, producing both functional and structural adaptations and reducing systemic inflammation [173, 174]. In this sense, the increased shear stress during exercise contributes to an antiatherogenic phenotype of arterial ECs [175], characterized by an increase in the NO pathway [176].

Current guidelines recommend weekly practice of at least 150 minutes of moderate-intense activity or 75 minutes of aerobic intense activity or an equivalent combination of both, with a minimum of 10 minutes per session to achieve substantial CV benefits, as there is a dose-response relationship [1, 151] Resistance exercise is also recommended, as it helps to control glycaemia and blood pressure. However, in people unable to fulfill current recommendations, physical activity should be adapted according to individual's capacities, since short sessions seem to be also beneficial [151].

In a recent meta-analysis of type 1 diabetes and physical training on CV risk factors, exercise reduced glycated haemoglobin, daily insulin requirements, and total cholesterol [177]. All types of exercise tended to reduce body fat or weight but both aerobic and combined exercises improved cardiorespiratory fitness. Furthermore, combined exercise significantly reduced diastolic blood pressure [178]. In the MESA study, 1970 adults and older individuals without CVD were followed up to 6 years to evaluate the relationship between inflammatory biomarkers and exercise. The study demonstrated an inverse relationship between physical activity and IL-6, leptin, resistin, and TNF-*α* levels independently of CV risk factors. Moreover, physical activity increased adiponectin levels, which were related with a reduction in central adiposity [179].

Cai et al. [180] evaluated the effects of 12 weeks of swimming on atherosclerosis ApoE^−/−^ and C57BL/6 mouse models fed with high-fat diet (HFD). Nontrained atherosclerotic mice had reduced HDL levels and increased body weight, total cholesterol, LDL, free fatty acid, insulin resistance, fasting plasma glucose, and insulin levels. Furthermore, they also had reduced miR492 and increased resistin expression in the aortic endothelium. Moreover, swimming delayed the severity of atherosclerosis and insulin resistance by increasing miR492 and decreasing resistin expression. Swimming decreased body weight and prevented metabolic alterations induced by HFD. After 12 weeks, histological changes of atherosclerosis presented in nontrained HFD mice were milder in the trained group.

In summary, exercise enhances endothelial function, prevents oxidative stress and inflammation, reduces adrenergic tone and the levels of ET-1, TG, apoB, and LDL, and increases HDL [181].

### 5.3. Diet

Elevated consumption of trans-free fatty fats, red meat, sugar-sweetened soft drinks, and salt increases the risk of CVD [151].The 2019 ACC/AHA Guideline on the primary prevention of cardiovascular disease recommends to reduce the intake of such products.

Trimethylamine-N-oxide (TMAO), a metabolite of choline and L-carnitine found in red meat, promotes the formation of foam cells by increasing the expression of scavenger receptors on macrophages and reducing reverse cholesterol transport [182].

Trans fatty acids (TFAs) are unsaturated fat found in foods obtained from ruminants, such as dairy products and meat, although the most consumed TFAs are artificially produced by partial hydrogenation of vegetable oils mainly present in fast food. Artificial TFAs are associated with an increased risk of atherosclerosis and CV events [151, 183]. TFAs increase Lp(a), TG, and LDL and decrease LDL particle size and HDL levels. TFAs also increase proinflammatory cytokines and induce endothelial dysfunction and insulin resistance [183].

On the other hand, replacing animal proteins by plant proteins significantly reduced CV mortality. In the same line, the reduction of sodium intake, either as supplement or in food, reduces blood pressure and the incidence of CV events. In this sense, current guidelines recommend [1, 151] to follow a diet primarily composed of vegetables, fruits, legumes, nuts, whole grains, and fish and to replace saturated fat for monounsaturated (MUFA) and polyunsaturated (PUFA) fatty acids. It is recommended to avoid or minimize the intake of red and processed meats, dairy products, salt, sugar soft drinks and sweets, and trans unsaturated and saturated fats.

Healthy eating habits such as Mediterranean diet, primarily based on olive oil, cereals, vegetables, legume, fruits, and nuts, and moderate consumption of fish, poultry, and wine and low consumption of red meat, dairy products, and saturated fatty acids, have proven to be effective in reducing the incidence of major CV events in both primary [184] and secondary prevention [185]. The primary prevention Spanish multicenter and randomized trial PREDIMED (Prevención con Dieta Mediterránea) evaluated long-term effects of Mediterranean diet on high-risk people without cardiovascular disease for major cardiovascular events. The 7447 participants were assigned to receive a Mediterranean diet supplemented with either extravirgin olive oil or mixed nuts or a control diet with low fat intake recommendation. The latest report of this study found that the risk for major CV events at 5 years was lower in the groups receiving Mediterranean diet compared to control diet (3.6% for olive oil, 4.0% for mixed nuts, and 5.7% for control) [184], and such differences were greater in people with better adherence to Mediterranean diet [184, 185]. Furthermore, Mediterranean diet also improved CV risk factor control, inflammation, and oxidative stress [186]. Healthy effects of Mediterranean diet are related to the additive effects of its nutrients, as extensively reviewed Torres et al. [182]. In short, the beneficial effects of Mediterranean diet could be attributed to the anti-inflammatory and antioxidant effects of its compounds, acting synergistically to control risk factors and prevent against atherosclerosis. These compounds can modulate gene and protein expression of proatherogenic genes involved in first stages of atherosclerosis [186].

## 6. Treatment

### 6.1. Antiplatelet Therapy

Antiplatelet therapy is the cornerstone of CVD. The effectiveness of antiplatelet therapy lies on the control of platelet activation and chemokine release.

The accessibility and low cost of acetylsalicylic acid (AAS), coupled with old studies showing a decline in cardiovascular events [187–189], have made AAS the most widely prescribed drug for both primary and secondary CV prevention [190], despite the increased risk of major bleeding associated with its use [139, 187, 191, 192]. The effectiveness of AAS lies on the control of platelet activation and chemokine release. AAS irreversibly inhibits cyclooxygenase-1 (COX-1), suppressing the production of prostaglandins and TXA_2_ [139, 192–194]. Actually, AAS blocks platelet activation and aggregation by reducing the expression of surface receptors GPIIb/IIIa and P-selectin and the release of chemokines such as CX3CL1 or fractalkine, which is involved in cell adhesion [195, 196], and PF4 and SDF-1 from exosomes, which are implicated in oxLDL uptake from macrophages and, therefore, foam cell formation [139]. While AAS is still indicated for secondary prevention in high-risk patients [1, 151, 193], its efficacy in healthy individuals without known atherosclerosis is being questioned [196].

While some authors suggest a therapeutic window for aspirin in relation to body weight and propose that a weight-adjusted dose could be beneficial [197], concerns exist about the inconsistencies surrounding the correlation between body weight and cardioprotective doses [190]. In addition, the optimal dose and dosing interval is yet to be determined [193, 198] and the risk of bleeding is of major concern [199]. However, with the development of more efficacious drugs for the management of major CV risk factors and the adoption of healthy life habits that have reduced the incidence of CV events, the widespread use of AAS for primary prevention is now questioned [198] and is no longer widely recommended [1, 151]. In this sense, recent trials assess the current effectiveness of aspirin for primary prevention. The aspirin to reduce risk of initial vascular events (ARRIVE) trial [200] randomized 12546 men and women over 55 and 60 years of age, respectively, without diabetes and a 10-year moderate CV-calculated risk, into two groups: one receiving 100 mg of aspirin per day and the other receiving a placebo. The 60-month follow-up period revealed a lower global rate of CV events than expected (4.3 for aspirin and 4.5 for the placebo group), without differences between the groups. The incidence of myocardial infarction was lower in the aspirin group only in the per protocol analysis, without any further differences in other adverse events being observed in either intention-to-treat or per protocol analysis. The ARRIVE trial results could be in part attributed to a better management of risk factors, as 43% of the participants were taking statins and 75% antihypertensives. Besides, the incidence of gastrointestinal bleeding was higher in the aspirin group (61 vs 29 patients, *p* = 0.007). In the ASCEND (A Study of Cardiovascular Events in Diabetes) trial [201], 15480 diabetic participants over 40 years old without CVD were randomized to 100 mg daily of aspirin or placebo. As in the ARRIVE trial, a high percentage of patients were under treatment with statins and antihypertensives. After a 7.4-year follow-up period, there were 12% less serious vascular events in the aspirin group (8.5% vs 9.6%, for aspirin and placebo groups, respectively, *p* = 0.01). However, the 29% increase of major bleeding events in the aspirin group (4.1% vs 3.2% for aspirin and placebo groups, respectively, *p* = 0.003%) offsets the beneficial effects of aspirin on these persons. Finally, in the Aspirin in Reducing Events in the Elderly (ASPREE) trial [202], 19114 aged participants without a history of cardiovascular disease were randomized to 100 mg daily of aspirin or placebo. Thirty-four percent of patients in both groups were taking statins. After 4.7 years of follow-up, no differences were found between groups in terms of incidence of CV events, though the aspirin group showed a significantly increased rate of major bleeding.

In a recent meta-analysis [203] involving 164225 participants with a mean baseline risk of primary CV events of 9.2%, people receiving AAS as a preventive treatment had an absolute risk reduction of 0.38% for unfavourable cardiovascular outcomes but showed an increased risk of major bleeding of 0.47% compared with untreated participants. Another meta-analysis of 11 trials of AAS for primary prevention (around 157000 participants) did not find a significant risk reduction in mortality but detected a significantly increased incidence of major bleeding (mean relative risk of 1.47), including diabetic and CV high-risk patients [198]. All in all, results from latest trials, guidelines, and recent meta-analyses do not support the systematic use of aspirin for primary prevention of CVD and recommend a careful individual evaluation of risks and benefits. Thienopyridines such as clopidogrel, ticagrelor, and prasugrel are currently used in clinical practice for secondary prevention of CVD [194]. They are selective antagonists of the ADP-induced activation of the P2Y12 receptor, interfering in platelet activation and aggregation. Furthermore, these antagonists reduce CD40L and RANTES plasma levels [192, 204]. However, their role in primary prevention is not supported by the literature, as reviewed by other authors [194, 205].

### 6.2. Statins

Lipid-lowering measures reduce inflammation and have immunomodulatory properties [206]. The most effective drugs currently used for hyperlipidaemia are statins. By inhibiting the 3-hydroxy-methylglutaryl coenzyme A (HMG-CoA) reductase, statins block the conversion of 3-hydroxy-methylglutaryl coenzyme A in L-mevalonate, a precursor of cholesterol synthesis [207]. Statins had proven to significantly reduce the risk of major cardiovascular events [145]. In this sense, virtual histology intravascular ultrasound studies showed significant plaque size reduction in patients with atherosclerosis taking statins [208]. Besides lowering LDL cholesterol levels, benefits of statins are related to their pleiotropic actions [139, 207, 209], mainly through their anti-inflammatory effects [206, 210], which were extensively reviewed elsewhere [139, 207, 209]. In this sense, statins reduce the levels of proinflammatory chemokines (MCP1, RANTES) and cytokines (IL-1*β*, TNF-*α*, IL-6, IL-8, and CD40L), adhesion molecules such as P-selectin and ICAM-1, NF-*κ*B transcription factor, and monocyte activation. They also reduce oxidative stress, by decreasing Nox and O_2_^−^ formation and LDL oxidation and by increasing free radical scavenging. Furthermore, statins upregulate eNOS activity and NO levels through different pathways, such as by improving BH4 bioavailability in patients with coronary artery disease [211] or by posttranscriptional modulation [212]. In addition, statins increase PGI_2_ levels and prevent platelet aggregation and activation [207]. Statins give stability to the plaque preventing further complications, by stimulating collagen synthesis and decreasing MMPs, macrophages, and inflammatory cells in the atheroma [207].

Furthermore, via PPAR*γ* activation, statins facilitate the conversion of monocytes into M2 macrophages in peripheral blood mononuclear cells from patients treated with rosuvastatin [213], promoting the anti-inflammatory macrophage phenotype, thus decreasing markers of inflammation and oxidative stress. In a model of kidney disease, simvastatin attenuated Ang II-induced inflammation and oxidative stress in human mesangial cells [214]. Moreover, the combination of statins and PPAR*γ* agonists has additive beneficial effects on atheroma regression in a rabbit model of atherosclerosis [215]. Importantly, statins were proven to significantly reduce CRP levels and thus the risk of incidence of major cardiovascular events [145, 216, 217].

Finally, six weeks of statins increased Treg, Foxp3a, IL-10, IL-4, and TGF-*β* and decreased IL-1*β*, IL-17, and IFN-*γ* in carotid atherosclerotic plaques in ApoE^−/−^ mice [218]. Moreover, statins increased Treg cells in cultured peripheral blood mononuclear cells from patients with acute atherosclerotic coronary syndrome [218].

By all these mechanisms, statins prevent the development and progress of atherosclerosis, acting from the earliest phases of the process, preventing endothelial damage and thus platelet-endothelium interaction. These effects may vary according to the dose and statin type [219, 220].

### 6.3. PCSK9 Inhibitors

Recent studies are addressing the effects of proprotein convertase subtilisin/kexin type 9 (PCSK9) inhibitors as an add-on therapy for further reducing LDL levels and increasing protection against major cardiovascular events [221]. PCSK9 is mainly expressed and secreted in the liver and regulated by cellular cholesterol levels [222]. Other organs, such as the intestine, kidney, and central nervous system, express PCSK9 [222]. Circulating PCSK9 binds to the LDL receptor, which is internalized and degraded by lysosomes, impeding receptor recycling, which results in increased serum LDL concentrations [221, 223, 224]. Moreover, treatment with PCSK9 significantly increases apoB secretion in human enterocytes [225]. Besides, PCSK9 also binds the VLDL receptor and ApoE receptor 2, thus interfering VLDL and apolipoprotein metabolism [221, 223]. PCSK9 plasma levels are increased in aged people, postmenopausal women, and patients with elevated body mass index, hypercholesterolemia, diabetes, and high CPR levels [226]. Moreover, elevated plasma levels of PCSK9 were found to be associated to the development of acute organ failure in patients with sepsis [227]. In the same way, PCSK9 levels were positively correlated with the severity of coronary lesions in patients with acute coronary disease [228], supporting the relationship between systemic inflammation and PCSK9 levels [229]. Familial hypercholesterolemia seems to be linked to gain-of-function PCSK9 mutations [223, 224, 230], and the inhibition of PCSK9 reduces LDL levels and the risk of CV events in these patients [231]. On the contrary, the loss-of-function mutations were associated with lower levels of LDL and incidence of CVD [221]. Therefore, the blockade of PCSK9 prevents LDL receptor degradation and promotes LDL clearance. The current PCSK9 inhibitors approved by FDA (US Food and Drug Administration) and EMA (European Medicines Agency) are the human monoclonal antibodies alirocumab and evolocumab, but other pharmaceutical approaches are currently under research [221]. Evolocumab reduced the volume of the atheroma plaque in patients with angiographic coronary artery disease [232]. Moreover, it reduces the levels of LDL, apolipoprotein B, lipoprotein(a), and triglycerides [221, 233–235]. In patients with atherosclerotic CVD and high levels of LDL in treatment with statins, evolocumab further reduced LDL levels (59% compared vs placebo) and the risk of major cardiovascular events by 15-20% [236]. Alirocumab also reduces the risk of major CV events and death in patients with acute coronary artery disease and hypercholesterolemia [237, 238]. Clinical studies demonstrated that PCSK9 inhibitors reduce the incidence of major cardiovascular events in patients with CVD but not cardiovascular mortality [239]. Ongoing studies will assess the effectiveness of these drugs on patients with acute cardiovascular events such as acute coronary syndrome, exploring not only the degree of LDL lowering but also their effect on inflammatory markers, platelet function, or plaque composition [240].

Results from in vivo and in vitro studies show that PCSK9 has proinflammatory effects by both LDL receptor-dependent and independent mechanisms [241]. Lipopolysaccharide- (LPS-) treated PCSK9 knockout mice produced lower levels of inflammatory cytokines TNF-*α*, IL-6, MCP-1, and macrophage inflammatory protein 2 (MIP-2) [242] and IL-1*β* [243]. Furthermore, the presence of the genetic variant of PCSK9 loss-of-function in patients with septic shock and in healthy subjects after LPS administration showed a decreased inflammatory cytokine response such as TNF*α*, IL-6, MCP-1, and IL-8 [242]. On the contrary, PCSK9 overexpression in an ApoE^−/−^ mouse model increased TNF-*α* and IL-1*β* expression and reduced anti-inflammatory markers IL-10 and Arg1 by an LDL receptor-dependent mechanism [244].

PCSK9 is expressed in monocyte/macrophages, VSM cells, and ECs [243–245]. PCSK9 inhibition reduces the monocyte proinflammatory phenotype in hypercholesterolemic mice by lowering LDL, resulting in decreased atherosclerotic lesion size and improved plaque morphology [246]. Furthermore, it reduces monocyte CCR2 expression and migratory capacity and TNF-*α* levels in patients with familial hypercholesterolemia [245]. PCSK9 modifies plaque composition in ApoE^−/−^ mice, increasing monocyte infiltration and macrophage inflammation [244]. Culture cell of macrophages incubated with human PCSK9 increased mRNA levels of proinflammatory cytokines IL-1*β*, IL-6, and TNF-*α* and chemokines MCP-1 and CXCL2, both involved in monocyte recruitment in the plaque. Activation of the JAK and SREBP pathways and NF-*κ*B transcription factor seems to be involved in these responses [247].

PCSK9 is increased in VSM cells in human atheroma, promoted by vascular oxidative stress participating in cell damage [243, 248]. In VSM and ECs, both PCSK9 and LOX-1 regulate each other and inflammatory states upregulate LOX-1 and PCSK9 expression, which trigger or are triggered by mitochondrial ROS (mtROS) [248]. Furthermore, mtROS enhances mtDNA damage and PCSK9 expression in cultured VSM cells [249]. Moreover, inhibition of PCSK9 decreases LOX-1 and VCAM-1 expression in VSM cells [248] and LOX-1 depletion reduces PCSK9 and VCAM-1 expression, putting in evidence the bidirectional regulation between LOX-1 and PCSK9 [248]. Collectively, these studies highlight the potential role of PCSK9 inhibition in preventing the development and progress of atherosclerosis, targeting the first steps of the plaque formation.

### 6.4. Targeting Immunity and Inflammation

Another emerging atherosclerosis target is IL-1*β*, part of the IL-1 family, which are the cytokines most frequently involved in inflammatory processes through NF-*κ*B activation and the JNK and p38MAPK pathways [250]. IL-1 is an upstream pleiotropic proinflammatory cytokine that induces not only its own production but other signalling inflammatory pathways, such as IL-6 [152]. Its receptor contains a Toll-IL-1-receptor domain and plays a key role in the innate immune system. The main circulating form of IL-1 is IL-1*β*. It is synthesized as proIL-1*β*, which is transformed in the active form by caspase-1 activation by NLRP3 inflammasome [251]. IL-1*β* production is induced by several stimuli such as IL-1*α* and IL-1*β*, Toll-like receptor (TLR) agonists [251], and activators of the inflammasome such as cell debris, ischemia, or cholesterol crystals [252], amplifying the inflammatory response [253].

In a mouse model of atherosclerosis fed with high-fat diet, the lack of type I IL-1 receptor (IL-1R1) showed smaller atherosclerotic plaque development, reduced Nox4 mRNA, ROS production, and markers of inflammation, with an increased NO bioavailability [254]. Furthermore, IL-1 is expressed in human atherosclerotic plaques [255] and plays a key role in the formation, progression, and complications of the disease [255, 256]. Treatment of autoinflammatory diseases blocking IL-1*β* receptor results in anti-inflammatory effects [257], a response also seen in other chronic diseases such as type II diabetes [258] or heart failure [259].

Anakinra, a competitive IL-1R1 blocker, is approved for the treatment of rheumatoid arthritis and other acute and chronic autoinflammatory diseases such as Still disease or cryopyrin-associated periodic syndromes (CAPS) [257, 260]. Furthermore, anakinra reduced the CRP levels and the incidence of heart failure in patients with acute myocardial infarction [256]. Despite the higher incidence of infections, anakinra has a safety profile, being the most used IL-1 blocker to date [253].

Canakinumab is a specific monoclonal IL-1*β* antibody that prevents the activation of the IL-1 receptor [253]. Canakinumab is currently approved for the treatment of autoinflammatory diseases such as CAPS, periodic fever syndromes, familial Mediterranean fever, and gouty arthritis among other diseases [253]. Canakinumab reduced CRP, IL-6, and fibrinogen levels in patients with diabetes mellitus and high cardiovascular risk [261]. The Canakinumab Anti-inflammatory Thrombosis Outcomes Study (CANTOS), a randomized, double-blind, placebo control trial [146], demonstrated that canakinumab significantly reduced hsCRP and IL-6 levels and the incidence of recurrent cardiovascular events in patients with a history of myocardial infarction. These effects were independent of cholesterol levels. However, canakinumab did not modify all-cause mortality, and, in addition, treated patients had increased incidence of neutropenia and severe infections and thrombocytopenia.

Results from clinical trials [146, 261] prove that IL-1*β* blockade is a promising therapy to halt the development and progression of atherosclerotic plaque, thus preventing cardiovascular diseases.

IL-6 plays a key role in propagating the inflammatory response and consequently the development of atherosclerosis [262]. IL-6 induces acute-phase reactions and cellular and humoral responses to the acute and chronic inflammatory states. Furthermore, IL-6, which activates ECs, is involved in leucocyte recruitment and induces the release of IL-8, MCP-1, and adhesion molecules. Thus, the inhibition of IL-6 could prevent the development of inflammatory diseases [262, 263]. IL-6 levels could be a potential biomarker of the early stages of atherosclerosis and a predictor of the development of cardiovascular diseases in healthy subjects [264–266]. The inhibition of IL-6 with tocilizumab prevented the increase of PCSK9 in patients with myocardial infarction [267], and inflammatory response in myocardial infarction patients underwent percutaneous coronary intervention [268]. However, the treatment with tocilizumab increased the LDL and triglyceride levels in rheumatoid arthritis patients [269]. In this regard, further studies are needed to evaluate the efficacy and safety of IL-6 blockade in preventing atherosclerosis and the development of CVD.

IL-8 is a chemotactic cytokine for neutrophils and lymphocytes produced by macrophages and is highly expressed within human atherosclerotic lesions. IL-8 promotes angiogenesis and plaque formation, and that is why it is considered a key regulator in the atherosclerotic process [270]. Lu et al. [271] found that the IL-8 monoclonal antibody can be coupled to stable biological microbubbles and used for detecting atherosclerotic plaque in a rabbit model of atherosclerosis. Lin et al. [272] identified IL-8 as the only cytokine increased in human mesenchymal stem cells (hMSCs) upon exposure to oxLDL. Using the condition medium derived from oxLDL-treated hMSCs for their experiments, they demonstrated that this medium reversed the inhibitory effect of oxLDL on the Akt/eNOS pathway in human umbilical vein endothelial cells (HUVECs) in a dose-dependent manner. Moreover, this beneficial effect was blocked by adding IL-8 neutralization antibodies, thus indicating that activation of the endothelial Akt/eNOS pathway by IL-8/MIP-2 is involved in the mechanism of MSCs for ameliorating atherosclerosis. However, further investigation is needed in order to assess the use of IL-8 antibodies to treat atherosclerosis.

Toll-like receptors play a central role in macrophage activation, inducing leukocyte recruitment and enhancing MMP expression within atherosclerotic lesions, providing a strong link between local innate and adaptive immunity. Toll-like receptor 9 (TLR9) is expressed in immune system cells and plays a pivotal role in atherosclerosis. Fukuda et al. [273] demonstrated that Ang II treatment increased the level of plasma cell-free DNA (cfDNA), an endogenous ligand for TLR9, in Ang II-infused ApoE^−/−^ mice. They showed that TLR9 activation promotes inflammation and activation of macrophages, partially via p38 MAPK. Moreover, they demonstrated that circulating cfDNA levels in the coronary artery in patients with acute myocardial infarction were associated with vulnerable features of atherosclerotic plaques, suggesting the participation of cfDNA-TLR9 signalling in the development of vascular inflammation and atherosclerosis. Zhang et al. reported that curcumin protects against atherosclerosis, partially by inhibiting TLR4 [274]. Curcumin reduced IL-1*β*, TNF-*α*, VCAM-1, ICAM-1, NF-*κ*B activity, and TLR4 expression induced by lipopolysaccharide, thus suppressing atherosclerosis development in ApoE^−/−^ mice. However, some Toll-like receptors have a protective role in atherosclerosis [275].

Targeting T cells, whether by enhancing Treg activity or reducing Th1, could be a promising approach to prevent atherosclerosis at early stages [47, 48]. In this sense, different experimental studies using hypercholesterolemic mice showed that decreasing Treg or IL-10 increased atherosclerosis and increased Th1. Conversely, overproduction of IL-10 and TGF-*β* levels reduced the development of atherosclerosis [48]. Targeting T cell effector functions, such as blocking proinflammatory cytokines, could be an interesting approach to prevent or reduce atherosclerosis. However, immunosuppression increases the risk of infections. Targeting Treg cells, by increasing IL-2, immunization, or converting effector T cells into Treg cells is another approach to prevent against atherosclerosis [48].

As we have explained earlier, the activation of LOX-1 is crucial to the development of atherosclerosis; hence, antibodies to LOX-1 are a promising treatment for early atherosclerosis. An interesting review by Pothineni et al. describes the possible therapeutic use of LOX-1 modulation by means of natural and synthetic compounds [276]. Actually, the inhibition of LOX-1 activity by using monoclonal antibodies is under research. Using chickens immunized with recombinant human LOX-1, Iwamoto et al. [277] generated 53 monoclonal antibodies that were shown to decrease oxLDL uptake.

Vaidya et al. recently reviewed past and current trials targeting atherosclerosis and inflammation [278]. Ongoing studies are evaluating the usefulness of colchicine as an adjuvant drug for the treatment of atherosclerosis based on its anti-inflammatory properties. Colchicine is a safe and widely available drug used for the treatment of gouty arthritis, pericarditis, and familial Mediterranean fever [278]. As reviewed recently, besides inhibiting neutrophil function and activation, colchicine blocks NLRP3 inflammasome activation, thus reducing the production of proinflammatory cytokines IL-1*β* and IL-18 by monocytes [103]. Colchicine reduced hsCRP levels in patients with coronary artery disease [278–280] and improved plaque morphology [278] and endothelial function in patients with leukocyte activation [279]. Moreover, low dose of colchicine reduced the incidence of cardiovascular events in patients with stable coronary artery disease [281].

Methotrexate and TNF blockade reduce the risk of CVD in patients with autoimmune diseases [282]. However, low-dose of methotrexate failed to lower the incidence of cardiovascular events in patients with previous coronary artery disease [283].

An alternative strategy to target inflammation is enhancing physiological anti-inflammatory mechanisms; accordingly, stem cells represent a novel approach to regulate immunity and inflammation.

### 6.5. Stem Cells in Atherosclerosis

Somatic stem cells play a central role in regenerative processes. Stem/progenitor cell therapies have been proved to be safe, viable, and effective in some early-phase clinical trials. However, there are still few late-phase clinical trials. Fujita and Kawamoto provide an overview of the preclinical and clinical reports demonstrating the usefulness and current limitations of cell-based therapies [284].

The severity of injury can sometimes overcome the regenerative response of stem cells, like that in chronic diseases such as atherosclerosis. Until now, the efficacy of stem cell-based therapies for atherosclerosis has been inconsistent [285].

Due to their capacity for immunomodulation, mesenchymal stem cells (MSCs), also named multipotent stromal cells, have evolved as promising therapeutic agents for atherosclerosis. Li et al. [286] have extensively reviewed the role of MSCs in animal models of atherosclerosis. MSC transplantation improves endothelial function, modulates cytokine secretion, promotes Treg cell function, decreases dyslipidaemia, and stabilizes vulnerable plaques during atherosclerosis development. Moreover, it affords benefits like low immunogenicity or inhibition of T cell responses [287]; hence, allogeneic MSC transplantation could be an attractive approach for treating atherosclerosis. MSCs exhibit atheroprotective effects on a model of atherosclerosis induced by high-fat diet in ApoE^−/−^ mice or LDL receptor knockout mice [286].

MSCs seem to be able to accelerate the repairing process of ECs. It has been shown that allogeneic bone marrow-derived MSC transplantation attenuates atherosclerosis by improving endothelial function. Lin et al. [272] studied the effects of MSCs on the atherosclerotic process in human and mouse ECs treated with oxLDL and in ApoE^−/−^ mice fed a high-fat diet. oxLDL inactivated the Akt/eNOS pathway, and coculture with human MSCs reversed these effects. Both the atherosclerotic plaque and endothelial dysfunction induced in ApoE^−/−^ mice fed a high-fat diet were ameliorated after systemic infusion with mouse MSCs. Moreover, MSCs have also been shown to significantly decrease VLDL plasma levels in MSC-treated mice after a 5-week treatment [288].

Numerous clinical and preclinical trials have suggested that MSC transplantation is safe [286]; however, the dosages for therapeutic application have not yet been determined. Much investigation remains to be performed in order to translate the current findings into clinical practice.

### 6.6. Endothelial Progenitor Cells

Endothelial progenitor cells (EPCs) are circulating mononuclear cells with the capacity to migrate, proliferate, and differentiate into mature ECs in conditions associated with endothelial injury to restore vascular integrity and function, a process named vascular repair. EPCs express surface markers, some shared with mature ECs, allowing EPCs to exert their function in a paracrine fashion. EPC migration and differentiation are a NO-dependent mechanism, through activation of AMPK pathways [289], also participating growth and angiogenic factors, cytokines, and hormones. EPC number and function are inversely related to the degree of inflammatory mediators, ROS, and the severity of atherosclerosis. In this sense, in a rabbit model fed with high-fat diet, physiologic ischemia training delayed the development of atherosclerosis as a result of the increase in EPC [290]. A healthy lifestyle and some CV drugs such as statins, antihypertensive, or antidiabetics, increase circulating EPC levels and function and explain the mechanisms by which EPC promotes vascular repair. EPC transplantation has the potential to repair injured endothelium and restore vascular function. For instance, EPC therapy in a hypertensive-hypercholesterolemic hamster model improved vascular function, suppressed the development of atherosclerosis, ameliorated metabolic and inflammatory profile, and mobilized endogenous EPC [291]. However, candidates to EPC autologous transplantation often have EPC-impaired number and function, thus making it difficult to isolate, cultivate, and proliferate their own EPCs. Furthermore, potential complications associated with embolization or uncontrolled growth limit this technique [289, 292, 293].

### 6.7. Antioxidant Therapy

Most of traditional antioxidant supplements fail to improve the incidence of CV events, probably because the cellular concentration of antioxidants is too low or the antioxidant therapy is initiated too late in the course of the disease [53].

Ang II is related to ROS production in that it increases O_2_^−^ formation and reduces antioxidant defenses and it is clearly involved in atherosclerosis [294]. Consequently, treatment with angiotensin-converting enzyme (ACE) inhibitors or Ang II receptor blockers (ARBs) can halt the development of atherosclerosis. Elevated levels of Ang II increase the expression of xanthine oxidase, thus contributing to vascular dysfunction [51].

Nomura et al. studied the effect of febuxostat, an inhibitor of xanthine oxidase, on ApoE^−/−^ mice. They found that macrophages and aortic ECs increased the expression of xanthine oxidase in the atherosclerotic plaque and that febuxostat suppressed plaque formation and reduced arterial ROS, improving endothelial function. Moreover, febuxostat inhibited inflammatory cytokine release in murine macrophages in vitro, demonstrating that xanthine oxidase plays a proinflammatory role in the atherosclerotic plaque and the potential therapeutic role of febuxostat [70].

Mitochondrial ROS are crucial in the development of atherosclerosis, and mitochondria-targeted antioxidants can be used as therapeutic agents. Mitochondrial ROS scavengers, such as mitoquinone, can diminish free radical formation without affecting mitochondrial oxidative phosphorylation. Mitoquinone has been proven to reduce macrophages within plaques in a mouse model of metabolic syndrome [295]. Karnewar et al. [296] synthesized a mitochondria-targeted esculetin (Mito-Esc) and demonstrated that Mito-Esc, but not natural esculetin treatment, inhibited H_2_O_2_^−^ and Ang II-induced cell death in human aortic ECs. In addition, Mito-Esc administration to ApoE^−/−^ mice alleviated Ang II-induced plaque formation, monocyte infiltration, and serum inflammatory cytokine levels. The mechanisms involved were related to the increase of NO production via AMPK-mediated eNOS phosphorylation and the enhancement of SIRT3 expression through AMPK activation and the resulting mitochondrial biogenesis.

Gene therapies targeting the overexpression of antioxidant systems, such as SOD or catalase, can reduce ROS levels and improve atherogenesis in ApoE^−/−^ mice [80]. However, while SOD1 overexpression alone may increase the extent of atherosclerosis; overexpression of both catalase and SOD reduced atherosclerosis [80].

Alp et al. used transgenic mice with endothelial-targeted overexpression of GTP cyclohydrolase I, which is the rate-limiting enzyme in BH4 synthesis, to study the effect of endothelial BH4 availability on atherosclerosis. Transgenic mice were crossed into an ApoE^−/−^ background and fed a high-fat diet for 16 weeks. The results showed that transgenic mice had higher BH4 and cGMP levels, lower endothelial O_2_^−^ production and eNOS uncoupling, improving NO-mediated vasodilation and a reduction in aortic root atherosclerotic plaque compared with ApoE^−/−^ controls [297]. Therefore, targeting overexpression of genes that increase BH4 bioavailability may reduce eNOS uncoupling, oxidative stress, and atherosclerosis progression.

miRNAs control gene expression through posttranscriptional regulation. At a vascular level, miRNAs are implicated in angiogenesis, oxidative stress, inflammation, and apoptosis. Therefore, anti-miRNA oligonucleotides and antisense oligonucleotides (ASOs) can be used as therapeutic targets by modulating gene expression. Mipomersen is a FDA-approved antisense oligonucleotide used in familial hypercholesterolemia that prevents the formation of apoB-100, decreasing its plasma levels, as well as LDL and total cholesterol levels [53]. In addition, nanoparticles or viral vectors can deliver small interfering RNAs (siRNAs) to silence molecules involved in endothelial activation [298].

### 6.8. Nanomedicine

The delivery of drugs by nanoparticles (NPs) that bind to a specific target, namely, targeted nanocarriers (NCs), is under research. The local target-driven approach would improve drug efficacy with lower adverse effects. As NP for atherosclerosis are supposed to be delivered by bloodstream, physical properties of both blow flow and blood cells should be considered. Inflammation and immunity are actively involved in the genesis and complications of atherosclerosis, and inflammatory biomarkers are independent risk factors for cardiovascular events [299]; thus, both components and biomarkers of the atherosclerotic process become of great help in targeting vascular drug delivery. The increased permeability of endothelium and plaque vascularity would allow NC penetration in the vascular wall [104], as recently proved in experimental models of atherosclerosis using lipid-polymer hybrid nanoparticle translocation and liposomal nanoparticles, as reviewed by Kelley et al. [104]. NP can be carriers of anti-inflammatory drugs such as celastrol, an NF-*κ*B inhibitor, as Allen et al. demonstrated in a model of atherosclerosis using Ldlr^−/−^ mice [300] or antibodies such as ICAM-1 to target endothelial adhesion proteins in the left anterior descending artery from a patient suspected of coronary heart disease [301]. Strategies for improving the vascular efficacy of nanocarriers are beyond the scope of this review [104], but questions such as NC degradation, inactivation, clearance, or migration should be considered. Of utmost importance is the ability of NC/NPs to cross the endothelial barrier for local delivery of the active drug [104].

On the other hand, NP sensitive to low pH or high RONS microenvironment have been proved to be effective for the treatment of atherosclerosis [302] and NP with antioxidant properties may improve endothelial dysfunction associated with atherosclerosis [56]. More studies are necessary to acquire a greater understanding of this emerging technology.

### 6.9. Epigenetic in Atherosclerosis

Epigenetic regulation includes any inheritable chromatin modification that controls gene expression, such as DNA methylation or histone posttranslational modifications. Once epigenetic modifications are established, these marks can serve to propagate cellular memory; however, these modifications can be reversed by drugs.

Notwithstanding a positive correlation between DNA methylation and the atherosclerotic lesion grade, both states of DNA hypomethylation and hypermethylation have been described in atherosclerosis [303]. In vitro studies have shown that treatment of HUVECs with oxLDL leads to methylation, resulting in endothelial inflammation [304]. Other studies in humans and mice have shown global DNA hypermethylation of cytosines in the context of CpGs as an accompanying feature of atherosclerosis [305]. In addition, proatherogenic-disturbed blood flow has a role in DNA methylation. Both in vitro human aortic ECs and in vivo porcine aorta exposed to disturbed flow present DNA hypermethylation and increased vascular inflammation [306], suggesting a potential role for DNA methylation in the progression of atherosclerosis. Accordingly, treatment with DNA methyltransferase inhibitors (DNMTi) may be promising therapeutics for atherosclerosis. Azacytidine and decitabine are DNMTis with FDA approval for the treatment of hematologic malignancies. Decitabine inhibits macrophage migration, reduces the expression of inflammatory genes in these macrophages, and suppresses endoplasmic reticulum stress in Ldlr^−/−^ mice [307]. Another DNMTi is RG108, which inhibits DNMT1 and DNMT3A, a DNA methyltransferase associated with coronary heart disease [308]. Reduced shear stress increases the expression of DNMT3A leading to DNA methylation of CpG islands within the Kruppel-like factor 4 (KLF4) promoter and suppression of KLF4 transcription in the porcine aorta and in cultured human aortic ECs. This effect can be reversed by DNMT inhibitors, such as azacytidine and RG108 [306]. Hydralazine, approved by the FDA for the treatment of hypertension, is a DNMTi with demonstrated anti-inflammatory effects on a C57/BL6 mouse model of Ang II infusion [309]. Further work is needed to determine whether treatment with DNMTi has effects at different stages of the disease.

Histone acetylation has an important role in the establishment and maintenance of transcription. Several studies have demonstrated the participation of histone acetyltransferases (HATs) and histone deacetylases (HDACs) in atherosclerosis, often with conflicting results. It is likely that histone acetylation by HATs has a proatherogenic role, regulated partially by inflammatory transcriptional pathways. For example, curcumin, a p300 HAT inhibitor, has an anti-inflammatory effect via downregulation of NF-*κ*B activity and also enhances cholesterol efflux in macrophages from both humans and mice [310]. MG149, another HAT inhibitor, has proved to be a potent inhibitor of the NF-*κ*B pathway [311]; hence, it could be an interesting candidate for further investigation. Other HAT inhibitors with specificity toward p300 HAT, such as garcinol or anacardic acid, are being used to investigate the role of histone acetylation.

HDACs play an important role in atherosclerosis development, for instance, by increasing the histone deacetylation of SM22*α*, which results in a VSM cell phenotype switch from a contractile to a secretory phenotype [312]. Some HDAC inhibitors, such as vorinostat (suberoylanilide hydroxamic acid), approved by the FDA for cancer therapy, have immunoregulatory properties and thus are good candidates for atherosclerosis treatment. In mice, a single oral administration of vorinostat reduced TNF-*α*, IL-1*β*, IL-6, and IFN-*γ* levels induced by LPS [313]. Valproate, a class I selective HDAC inhibitor, attenuates atherosclerosis in ApoE^−/−^ mice fed hyperglycaemic diet and decreases ER stress [314]. However, trichostatin A, another HDAC inhibitor, produces contrary results. It increases aortic root lesions and macrophage scavenger receptor CD36 and consequently the uptake of oxLDL in Ldlr^−/−^ mice [315].

Epigenetics is a potential tool for the future treatment of atherosclerosis; however, much work needs to be done to fully understand both epigenetic regulation in atherosclerosis and the potential effect of epigenetic inhibitors against plaque progression. The main therapeutic approaches are summarized in Table 1.

## 7. Conclusion

Cardiovascular diseases are the leading cause of morbidity and mortality in Western population. Atherosclerosis is a slow pathological process that begins in early life, and part of this process can be prevented by lifestyle modification. As such, detection of the early stages of atherosclerosis could be life- and cost-saving. The identification of biological markers of atherosclerosis is crucial for preventing the development, progression, and complications of the disease. Biomarkers of inflammation, oxidative stress, and miRNAs controlling LDL or HDL genesis can be useful for identifying subclinical atherosclerosis once accurate screening methods become available.

Oxidative stress leads to endothelial dysfunction, which in turn leads to inflammation. Moreover, activated platelets interacting with a damaged endothelium contribute to the proatherogenic effects. All these mechanisms are involved in plaque formation. Knowing the pathways implicated in this process will pave the way for the development of therapeutic approaches against early atherosclerosis. Currently, the main strategies to reduce the atheroma plaque are focused on antiplatelet therapy, lipid-lowering drugs, and anti-inflammatory and antioxidant measures. In this regard, statins are the most effective drugs so far targeting the main mechanisms involved in plaque formation but future treatments using PCSK9 inhibitors, IL-1R1 blockers, or nanocarriers sensitive to a high-RONS microenvironment are promising therapeutic approaches against early atherosclerosis.

## Figures and Tables

**Table 1:** Therapeutic approaches against atherosclerosis.

Intervention type	Experimental or clinical condition	Subject	Antiatherogenic effects
*Clinical studies in humans*			
Clopidogrel	Randomized, placebo-controlled study involving patients with coronary artery disease and chronic aspirin therapy (*n* = 113) [204]	Human	P2Y12 receptor antagonist ↓ CD40L and RANTES plasma levels
Atorvastatin	Internal mammary artery from patients scheduled for coronary artery bypass graft surgery (*n* = 492) [211]	Human	Improved vascular NO bioavailability ↓ the production of vascular O_2_^−^
Evolocumab	Prospective, nonrandomized study in patients with heterozygous familial hypercholesterolemia (*n* = 11) [235]	Human	Inhibited PCSK9 receptor ↓ LDL and apolipoprotein B levels
Anakinra	Double-blind randomized study in patients with rheumatoid arthritis (*n* = 23) [260]	Human	IL-1 receptor antagonist Improved vascular function ↓ IL-6 and ET-1
Canakinumab	Double-blind, multinational phase IIb trial in patients with well-controlled diabetes mellitus (*n* = 556) [146]	Human	Inhibited IL-1*β* ↓ CRP, IL-6, and fibrinogen levels
Tocilizumab	Double-blind, placebo-controlled trial in patients with non-ST elevation myocardial infarction (*n* = 117) [267]	Human	Inhibited IL-6 Prevented the increase of serum PCSK9 levels
Colchicine	Double-blind, randomized, placebo-controlled study in patients with coronary artery disease (*n* = 28) [279]	Human	↓ hsCRP levels Induced leukocyte activation
*Animal studies*			
Stem cells	ApoE^−/−^ mice [288]	Mouse	Anti-inflammatory effect by reduced IFN-*γ*, IL-6, and TNF-*α* expression ↓ VLDL, circulating monocytes, and serum CCL2 levels
Simvastatin	Rabbit model of atherosclerosis [215]	Rabbit	Induced atheroma regression ↓ MMP activity Pronounced reduction in plaque size with simvastatin plus selective PPAR*γ* agonist
Alirocumab	APOE^∗^3Leiden.CETP transgenic mice [246]	Mouse	Inhibited PCSK9 receptor ↓ TC and TG plasma levels Improved plaque morphology ↓ ICAM-1 and monocyte adhesion
Febuxostat	ApoE^−/−^ mice [70]	Mouse	Inhibited xanthine oxidase Suppressed plaque formation ↓ MCP-1, IL-1*α*, and IL-1*β* ↑ eNOS mRNA levels
Mitoquinone	ATM^+/+^/ApoE^–/–^ and ATM^+/–^/ApoE^–/–^ mice [295]	Mouse	↑ mitochondrial antioxidant Prevented the increase of adiposity, hypercholesterolemia, and hypertriglyceridemia ↓ macrophage content and cell proliferation within plaques
Mito-esc	ApoE^−/−^ mice [296]	Mouse	Reduced plaque in thoracic and abdominal aorta ↓ monocyte/macrophage infiltration, ICAM-1, and CD45.2 levels
PEG-*b*-PPS micelles	*Ldlr* ^−/−^ female mice and RAW blue cells [300]	Mouse	Celastrol-loaded micelles reduced NF-*κ*B signalling and TNF-*α* secretion ↓inflammatory cells such neutrophils, monocytes and natural killer cells, and plaque area
Decitabine	*Ldlr^−/−^ mice* [307]	Mouse	Inhibited DNA methylation Downregulated expression of inflammatory genes (TNF-*α*, IL-6, IL-1*β*, and iNOS) ↓ macrophage migration and infiltration into atherosclerotic plaques
Hydralazine	C57/BL6 mouse model of Ang II infusion [309]	Mouse	Inhibited DNA methylation Blocked Ang II-induced fibrosis ↓ inflammatory cell infiltration and proinflammatory cytokine expression
Curcumin	ApoE^–/–^ mice [274]	Mouse	Reduced TLR4 expression and macrophage infiltration in atherosclerotic plaques ↓ TNF-*α*, IL-1*β*, VCAM-1, and ICAM-1 expression and plasma levels, and NF-*κ*B activity
Vorinostat	BALB/c mice [313]	Mouse	Inhibited HDACs Reduction of circulating TNF-*α*, IL-1*β*, IL-6, and IFN-*γ* induced by lipopolysaccharide
Valproate	Hyperglycaemic ApoE^−/−^ mice [314]	Mouse	Inhibited HDACs Attenuated endoplasmic reticulum stress response genes Decreased in cross-sectional lesion area of atherosclerotic lesion
*Cell culture studies*			
Aspirin	Platelets from healthy patients (*n* = 27) [195]	Human	Inhibited COX-1 ↓ the expression of platelet receptors (GPIIb/IIIa, P-selectin) and natural killer cell markers (CD107a and CD63)
Simvastatin	Cell culture of mesangial cells [214]	Human	↓ Ang II-induced inflammation and oxidative stress via COX-2, PPAR*γ*, NF-*κ*B, Nox, and PKC
Rosuvastatin	Cell culture of peripheral blood mononuclear cells [213]	Human	Promoted M2 macrophage phenotype ↑ PPAR*γ* mRNA expression ↓ TNF-*α* and MCP-1 levels
Mito-esc	Human aortic endothelial cells [296]	Human	Inhibited H_2_O_2_ and Ang II-induced cell death Promoted mitochondrial biogenesis by enhancing SIRT3 expression
Azacytidine and RG108	Human aortic endothelial cells [306]	Human	Inhibited DNMT3A Restored KLF4 pre-mRNA to undisturbed flow levels

↑: increased; ↓: decreased; NO: nitric oxide; PCSK9: proprotein convertase subtilisin/kexin type 9; LDL: low-density lipoprotein; IL: interleukin; ET: endothelin; hsCRP: high-sensitivity C-reactive protein; TNF-*α*: tumour necrosis factor *α*; VLDL: very low-density lipoprotein; MMPs: matrix metalloproteinases; ICAM-1: intercellular adhesion molecule 1; MCP-1: monocyte chemotactic protein-1; eNOS: endothelial nitric oxide synthase; NF-*κ*B: nuclear factor *κ*B; iNOS: inducible nitric oxide synthase; Ang II: angiotensin-II; TLR4: Toll-like receptor 4; VCAM-1: vascular cell adhesion molecule 1; HDAC: histone deacetylases; Nox: NADPH oxidases; PKC: protein kinase C; DNMT3A: DNA methyltransferase 3A.
